# Therapeutic targeting of BCL-2 during CART cell production augments potency through non-apoptotic adaptive changes

**DOI:** 10.1038/s41392-026-02655-y

**Published:** 2026-04-27

**Authors:** Nada S. Aboelella, Ryan Park, Erting Tang, Nicholas Asby, Joshua D. Ho, Tony Pan, Sidney Wang, Lishi Xie, Justin P. Kline, Peter A. Riedell, Jun Huang, James L. LaBelle

**Affiliations:** 1 Section of Hematology/Oncology, Department of Pediatrics, University of Chicago, Chicago, IL USA; 2 Pritzker School of Molecular Engineering, University of Chicago, Chicago, IL USA; 3 Section Hematology/Oncology, Department of Medicine, University of Chicago, Chicago, IL USA

**Keywords:** Immunotherapy, Immunological techniques, Target validation

## Abstract

Targeting the BCL-2 family of proteins, key regulators of cellular apoptosis, with BH3-mimetics has been a major therapeutic goal to overcome cancer cell death resistance. However, beyond their canonical roles in apoptosis, BCL-2 proteins also play vital roles in cellular metabolism, signaling, and, increasingly, immune cell regulation. T cells in particular depend heavily on BCL-2 family proteins during ontogeny and maintenance, yet the broader consequences of pharmacologically inhibiting anti-apoptotic BCL-2 proteins in T cells remain underexplored. Our group has previously demonstrated that BCL-2 inhibition with the BH3-mimetic venetoclax induces gene expression and plasticity changes in murine T cells. Building upon this, we here investigate whether venetoclax-driven T cell reprogramming can be leveraged to enhance adoptive cell therapies, specifically using chimeric antigen receptor (CAR) T cells targeting CD19 as a model system. Our findings reveal that venetoclax treatment during ex vivo expansion of CART cells, prepared from T cells from healthy donors or chemotherapeutically pretreated patients, potently augments antitumor efficacy in a BCL-2-dependent manner. Transcriptomic and functional analyses demonstrate that venetoclax reprograms CART cells by regulating signaling pathways (e.g., IL-2/STAT5 and PI3K/AKT) and metabolic programs (e.g., OXPHOS and glycolysis), yielding CART cells with superior fitness and effector functionalities. These results highlight a novel therapeutic approach using anti-apoptotic drugging to enhance the performance of adoptive T cell therapies and support further examination of venetoclax and other BH3-mimetics as immune modulators.

## Introduction

Evading programmed cell death, or apoptosis, is one of the many hallmarks of cancer.^1^ One way in which this occurs is through alterations of BCL-2 family transcriptional and protein expression levels.^2–4^ To overcome this, BH3 mimetic small molecules targeting specific anti-apoptotic BCL-2 family members have become increasingly used as anti-cancer therapeutics alone or in combination with other chemotherapies.^5^ The BCL-2-specific BH3 mimetic, venetoclax, is the most clinically advanced compound in this drug class and is FDA-approved for patients with chronic lymphocytic leukemia (CLL), small lymphocytic lymphoma (SLL), and acute myeloid leukemia (AML).^6–11^ Understandably, most clinical studies determining venetoclax efficacy have focused on its direct apoptotic effect on malignant cells. However, little is known about the effects of venetoclax on bystander “normal” cells, such as immune cells, and T cells in particular, which are primarily responsible for anti-tumor immune responses.

T cells are highly reliant on the BCL-2 family during development, activation, contraction, and homeostasis.^12,13^ Many of the studies proving the reliance of T cells on BCL-2 proteins have been performed using gene knock-out animal models where T cells often still develop, but may do so with alterations in their differentiation, phenotype, and function.^12,14–17^ This is largely due to redundancy among BCL-2 family members in regulating cell death in T cells despite the genomic loss of one family member.^18^ Understanding how this compensation arises in individual immune cell populations can be partially addressed using conditional gene knockout or overexpression models, but these can be confounded by the cell type-specific knockout or timing of cell death evaluation.^19^ Additionally, these models represent the absence or overreliance on critical BCL-2 family protein:protein interactions (PPIs) rather than their therapeutic disruption at natural protein levels. In fact, early work using microarray transcriptional analyses revealed that BCL-2 family members undergo dynamic changes soon after T cell activation, suggesting that coordinated alterations in the BCL-2 family network are critical for normal T cell effector signaling and function.^20^ How BH3-mimetic-induced adjustment of this BCL-2 family interactome in normal T cells affects their function represents a new area of study. Emerging data support that drugging the BCL-2 protein in particular may have modulatory effects on T cells beyond affecting cell death homeostasis.

Venetoclax targeting of BCL-2 has been found to enhance antitumor efficacy in murine tumor models in combination with immune checkpoint blockade in a CD8^+^ T cell-dependent manner.^21^ T cell effector enhancement has also been measured in T cells from patients with AML who were treated with venetoclax.^22,23^ In addition, we recently demonstrated that BCL-2 blockade by venetoclax during ex vivo expansion of murine T cells results in significantly increased anti-apoptotic protein expression, heightened cell death resistance, and alterations in anti-apoptotic protein dependency patterns. Importantly, T cells expanded in the presence of venetoclax were also increasingly resistant to cell death caused by venetoclax itself.^24^ We also found that long-term in vivo treatment with venetoclax during T cell homeostatic expansion following stem cell transplantation results in adaptive transcriptional reprogramming within developing naïve T cells. Gene signatures reflected an activated-like state characterized by upregulation of JAK/STAT and downregulation of MAPK and FOXO signaling pathways.^24^ Additionally, we found that venetoclax induces BCL-2–dependent PI3K/AKT/FOXO signaling alterations in CD4^+^FOXP3^+^ Tregs, resulting in RORγ-mediated Th17-like plasticity changes that synergize with anti-PD-1 checkpoint blockade to immunologically eradicate tumors.^25^ Whether such alterations can be directly leveraged to improve the effector function of adoptively transferred human T cell therapies, such as chimeric antigen receptor (CAR) T cells, is unknown but of potentially high clinical impact.

Combining BH3 mimetic treatment with CART cells has been examined with the intent of priming tumor cells for CART cell-mediated killing.^26–29^ These works found that overexpression of anti-apoptotic proteins, particularly BCL-2 and BCL-X_L_, intrinsically enhanced CART efficacy against xenograft models of leukemia and lymphoma. This was determined to be the result of greater CART expansion, reduced exhaustion, and prolonged in vivo CART survival.^27,28^ However, these studies did not directly examine whether these proteins may also have druggable intrinsic immune modulatory properties. In our study, we find that therapeutic BCL-2 targeting with venetoclax during ex vivo expansion of human CD19CART cells induces an adaptive-like genetic, metabolic, and functional reprogramming resulting in CART cells that better resist cell death and exhaustion compared to vehicle-treated CART. These changes led to a significant improvement in the antitumor efficacy of venetoclax-treated CD19CART driven by mechanisms that are BCL-2-dependent and involve STAT5/AKT signaling. Importantly, this ex vivo treatment approach improved the efficacy of CD19CART cells prepared from T cells isolated from heavily pretreated patients, irrespective of their baseline T cell composition and exhaustion patterns. These findings suggest that BCL-2 mediated PPI interactions represent immune modulatory nodes in CART cells and support further examination of the use of venetoclax to promote T cell effector function.

## Results

### Venetoclax directly enhances the effector function of murine CD19CART cells

Based upon our previous work showing that T cells adapt to BCL-2 drugging through adaptive effector-like changes, we sought to determine if venetoclax could amplify the efficacy of adoptively expanded T cells using CART as a model system. To first test this, we used a well-described murine T cell model engineered to express a murine anti-CD19 CAR (mCD19CART).^30,31^ Here, CD45.1 transgenic mice received vehicle or venetoclax 50 mg/kg for 15 days (5 days/week for 3 weeks) in an effort to provide long-term BCL-2 drugging as tested in our previous murine studies.^24,25^ T cells from these animals were isolated, activated, and transduced to express anti-murine CD19CAR and expanded ex vivo in the presence or absence of venetoclax (Supplementary Fig. S1a). We then examined the effector function of these mCD19CART through an in vitro cytotoxicity assay where differently treated mCD19CART were co-cultured with CD19^+^ murine B cell lymphoma (A20) at various effector to tumor (E:T) ratios. We found that administration of venetoclax either in vivo, ex vivo, or both resulted in enhanced antitumor mCD19CART killing of target cells compared to vehicle-treated CART (Supplementary Fig. S1b). However, ex vivo treatment with venetoclax of in vivo vehicle-treated cells during manufacturing resulted in the greatest enhancement of mCD19CART effector functionality (Supplementary Fig. S1c). Importantly, venetoclax did not affect CAR expression or the memory phenotype of the final CART product (Supplementary Fig. S1d, e). These results suggest that expansion of CART under BCL-2 blockade with venetoclax can increase CART-mediated tumor killing in a manner not related to apoptosis.

### BCL-2 blockade is compatible with human CART cell manufacturing

**Fig. 1:**
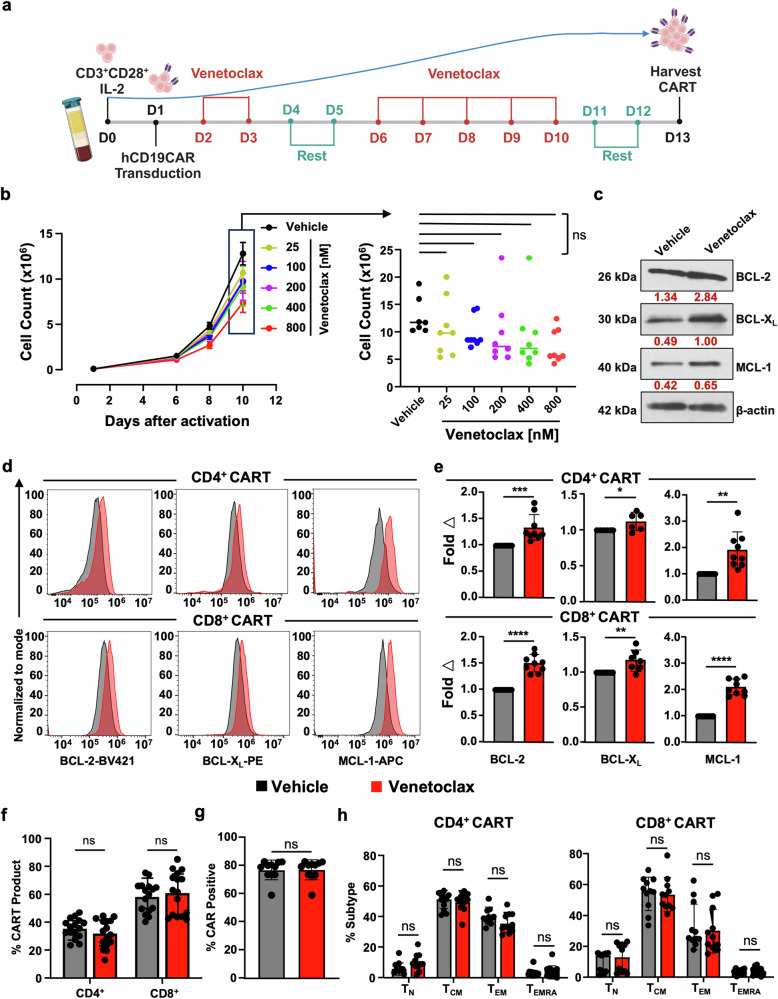
Venetoclax treatment is tolerated during CART expansion and induces upregulation of anti-apoptotic protein expression. **a** Schema of CD19CART (CART) production. T cells were isolated from PBMCs from healthy donors on day 0 and activated overnight with human anti-CD3/CD28 beads, followed by transduction with lentivirus designed to express hCD19CAR on day 1. On day 2, cells were either treated with vehicle (DMSO) or with the indicated doses of venetoclax daily until day 13. **b** CART treated with increasing doses of venetoclax shows no significant changes in T cell expansion compared to vehicle-treated CART. On left: proliferation curve indicating CART cell count for each treatment condition at different time points. On the right: comparison of CART cell counts on day 10 following treatment with increasing doses of venetoclax. **c**–**e** CART cells have increased anti-apoptotic protein expression upon expansion in the presence of venetoclax on day 13 of production. **c** BCL-2 family anti-apoptotic protein levels were evaluated in total vehicle vs venetoclax-treated CART by Western blot. Expression values (red) were normalized to the corresponding β-actin. **d** Protein expression measured using intracellular flow cytometric evaluation of BCL-2, BCL-X_L_, and MCL-1 in venetoclax-treated CART (red) and vehicle-treated CART (black). **e** BCL-2 family protein expression in venetoclax-treated CART normalized to expression in vehicle-treated CART. Venetoclax-treated CARTs show no significant differences in CART (**f**) CD4:CD8 ratio, **g** CAR^+^ expression, or **h** T cell memory phenotype. T_N_: naïve (CCR7^+^CD45RA^+^), T_CM_: central memory (CCR7^+^CD45RA^−^), T_EM_: effector memory (CCR7^−^CD45RA^−^), and T_EMRA_: terminally differentiated (CCR7^−^CD45RA^+^). Data are from at least three independent experiments from different healthy donors (*n* = 3) with two-three technical replicates in bar graphs and shown as mean ± SD. One-way ANOVA followed by Tukey’s multiple comparison tests was used for (**b**), unpaired Student’s *t*-test for (**e**–**g**), and two-way ANOVA followed by SIDAK’s multiple comparison tests for (**h**). ^*^P < 0.05, ^**^P < 0.01, ^***^P < 0.001, ^****^P < 0.0001, ns non-significant. Image (**a**) Created in BioRender

We next aimed to translate the previous findings to human CART cells. Given that BH3 mimetics were originally designed to induce target cell apoptosis,^32^ we wondered whether venetoclax treatment would negatively interfere with human CD19CART (hereafter referred to as CART) production by inducing cell death and limiting CART cell proliferation and expansion. To test this, following activation and CD19CAR lentivirus transduction, healthy donor CART were expanded with escalating doses of venetoclax (Fig. 1a). The dosing range chosen for treatment is equivalent to the plasma drug concentration measured in patients following standard dosing.^33^ Venetoclax resulted in a dose-dependent but non-statistically significant decrease in the number of cells in the final CART product (Fig. 1b). Taken together, this data suggests that venetoclax, by virtue of specific targeting of BCL-2, is a suitable BH3 mimetic to be tested and combined with CART manufacturing.

### Venetoclax-treated CART upregulates anti-apoptotic BCL-2 protein levels

We previously found that in vitro and in vivo treatment of murine CD4^+^, CD8^+^, and FOXP3^+^ regulatory T cells with venetoclax resulted in increased protein levels of BCL-2, BCL-X_L_, and MCL-1.^24^ Aligned with this phenomenon, CART expanded in the presence of venetoclax also showed upregulation of BCL-2, BCL-X_L_, and MCL-1 proteins in total CART (Fig. 1c) and similarly in CD4^+^ and CD8^+^ CART subsets (Fig. 1d, e). Like our initial studies in mCD19CART (Supplementary Fig. S1), venetoclax did not affect the CD4^+^/CD8^+^ ratio, CAR expression, or the memory population composition of the final CART product when compared to vehicle-treated cells (Fig. 1f–h). Collectively, these data indicate that ex vivo pressure on the apoptotic machinery via specific BCL-2 blockade results in compensatory upregulation of anti-apoptotic proteins without significantly affecting CART composition or expansion.

### Venetoclax enhances CART cytotoxicity in vitro

**Fig. 2:**
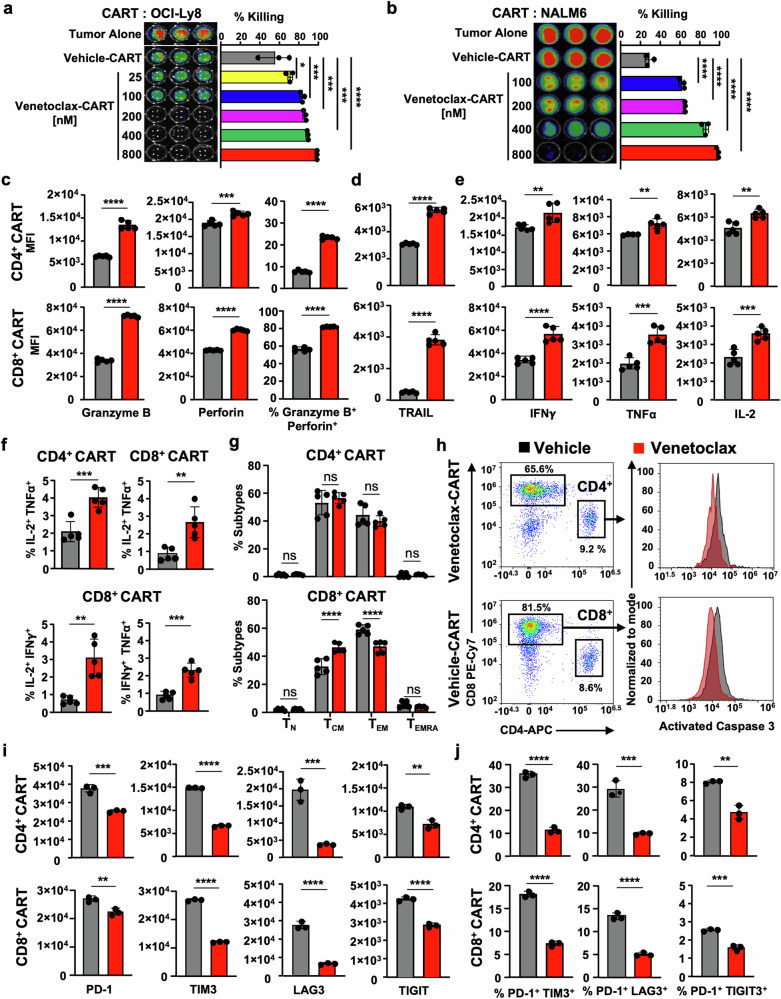
Venetoclax increases CART cytotoxicity in vitro, reflected in increased cytokine production, greater cell death resistance, and decreased exhaustion. Cytotoxicity of CART treated with vehicle or increasing doses of venetoclax against (**a**) CD19^+^ human DLBCL OCI-Ly8 and (**b**) CD19^+^ human acute lymphoblastic leukemia NALM6 cells using bioluminescent imaging quantification of remaining tumor cells after 48 h of co-culture at an E:T ratio of 1:5. Quantified results in adjacent bar graphs show that venetoclax dose-dependently enhances the anti-tumor efficacy of CART. **c** Venetoclax amplifies the killing function of CART via increased single and dual expression of granzyme B and perforin following stimulation with tumor target cells. **d**–**f** Venetoclax-treated CART upregulate expression of TNF-related apoptosis-inducing ligand (TRAIL) and have increased individual and co-expression of intracellular effector cytokines IFNγ, TNFα, and IL-2 following tumor stimulation. **g** Memory profiling shows no significant differences in CD4^+^ CART cell phenotype and indicates accumulation of T_CM_ in venetoclax-treated CD8^+^ CART while more T_EM_ are detected in vehicle-treated CD8^+^ CART. **h** Venetoclax-treated CART demonstrates longer persistence in vitro, marked by a reduced tendency to undergo apoptosis following encounter with OCI-Ly8 tumor cells. **i**, **j** Venetoclax-treated CART exhibit a less exhausted CART cell phenotype after two days of incubation with tumor cells. Data represent mean ± SD. Statistics for (**a**, **b**) one-way ANOVA with post-hoc Tukey’s tests were performed, unpaired Student’s *t*-test for (**c**–**f**, **i**–**j**), and two-way ANOVA with SIDAK’s multiple comparison tests for (**g**). **a**–**j** Results shown are from one representative experiment of at least three independent experiments with similar results using CART from three healthy donors. **g** Data are summarized from CART prepared from two healthy donors with two-three technical replicates in bar graphs and shown as mean ± SD. ^*^P < 0.05, ^**^P < 0.01, ^***^P < 0.001, and ^****^P < 0.0001, ns non-significant

Recent reports indicate that treating conventional or double-negative patient-derived T cells with venetoclax enhances their anti-leukemic activity through poorly understood mechanisms.^22^ Since our venetoclax-treated mCD19CART showed improved antitumor efficacy following ex vivo treatment (Supplementary Fig. S1), we wondered if venetoclax-treated human CART would have similarly enhanced activity even with their longer ex vivo expansion. To test this, we performed killing assays using vehicle or venetoclax-treated CART cocultured with the CD19^+^ expressing human diffuse large B cell lymphoma (DLBCL) cell line OCI-Ly8. Aligned with our previous findings, venetoclax-treated CART dose-dependently killed tumor cells more effectively than vehicle-treated CART (Fig. 2a). Importantly, venetoclax was not present in the cultures, thus ensuring that target cell death was not the result of direct BCL-2 drugging within tumor cells. Similarly, increased killing was observed against the CD19^+^ acute lymphoblastic leukemia cell line NALM6 (Fig. 2b). To ensure that venetoclax-treated CART induced antigen-specific killing, we repeated these cytotoxicity assays using CD19 null OCI-Ly8 tumor cells (OCI-Ly8.CD19KO) (Supplementary Fig. S2a). Venetoclax-treated CART failed to kill OCI-Ly8.CD19KO, indicating that CART-mediated killing was antigen-specific (Supplementary Fig. S2b). To test whether the enhanced cytotoxicity of venetoclax-treated CART was dependent upon having a certain co-stimulatory domain in the CAR construct, we compared killing by 4-1BB CD19CAR (used throughout this study) to an identical CD19CAR with a CD28 costimulatory domain. Additionally, to determine whether enhanced CART effector activity following venetoclax treatment is affected by different cytokines used during CART production, vehicle or venetoclax-treated CART were expanded in the presence of IL-2 (used throughout this study) or IL-7/IL-15. Venetoclax-treated CART induced similarly improved cytotoxicity in vitro regardless of which co-stimulatory molecule was incorporated into the CAR construct (Supplementary Fig. S2c), or the cytokine used during CART cell expansion (Supplementary Fig. S2d).

### Amplified T cell effector functions mediate venetoclax-treated CART enhanced cytotoxicity

To better understand the cytotoxic mechanisms responsible for venetoclax-mediated CART changes, we measured T cell effector cytokines, apoptosis, and exhaustion markers in venetoclax-treated CART following tumor antigen stimulation. Following co-culture with OCI-Ly8 cells, venetoclax-treated CART had greater expression of granzyme B, perforin, TRAIL, IFN-γ, TNF-α, and IL-2, all essential for CAR T-cell mediated cytotoxicity (Fig. 2c–f and Supplementary Fig. S3a). Cell death receptor/ligand pairs, including TRAIL/TRAIL-R1/R2, are known to play roles in CART cell effector killing through direct apoptosis induction of tumor cells.^34–36^ To test if TRAIL also played a significant role in venetoclax-treated CART-mediated killing, we performed neutralization experiments and found that antibody-mediated inhibition of TRAIL dose-dependently decreased venetoclax- and vehicle-treated CART efficacy (Supplementary Fig. S3b). We next wondered if venetoclax-related killing was reflected in an increased percentage of effector CART cells following tumor cell recognition. While the starting CART product from vehicle- and venetoclax-treated cells showed no differences in memory status (Fig. 1h), following tumor stimulation, there was a significant increase in the percentage of CD8^+^ central memory (T_CM_) and decreased percentage of CD8^+^ effector memory (T_EM_) cells within venetoclax-treated CART compared to the vehicle-treated product (Fig. 2g). Conversely, venetoclax-treated CD4^+^CART cells had no significant memory changes compared to vehicle-treated cells (Fig. 2g). Given these results, we next questioned if the increased anti-apoptotic protein expression measured in venetoclax-treated CART (Fig. 1c–e) may have led to less activation induced cell death (AICD), which can be associated with poor CART survival. Indeed, venetoclax-treated CART were more resistant to cell death following tumor target engagement compared to vehicle-treated cells, as reflected in less activated caspase 3 in these cells (Fig. 2h). The majority of these cells were CD8^+^ compared to CD4^+^ in both venetoclax- and vehicle-treated CART. However, venetoclax treatment led to a higher percentage of CD8^+^ cells compared to vehicle-treatment (81.5% vs 65.6%). In addition, compared to vehicle-treated cells, venetoclax-treated CART had significantly lower single and combined protein expression of canonical markers of T cell exhaustion, including PD-1, TIM3, LAG3, and TIGIT following co-culture with tumor cells (Fig. 2i, j). We wondered next if these results suggest that venetoclax-treated CART cells would kill target cells more efficiently and have prolonged serial killing ability. To test this, CART cells were incubated with OCI-Ly8 tumor cells at an effector:target (E:T) ratio of 1:1 and were serially challenged with tumor cells at the same E:T ratio until all tumor cells were killed. Venetoclax-treated CART cells were significantly better at killing tumor cells over time than vehicle-treated CART, while having similar proliferation in culture compared to vehicle-treated CART cells (Supplementary Fig. S3d). Thus, expansion of CART in the presence of therapeutic BCL-2 blockade improves their effector function, renders them more cell death resistant, and results in lower markers of exhaustion following interaction with target tumor cells.

### Venetoclax-enhanced antitumor activity of CART is BCL-2 dependent

**Fig. 3:**
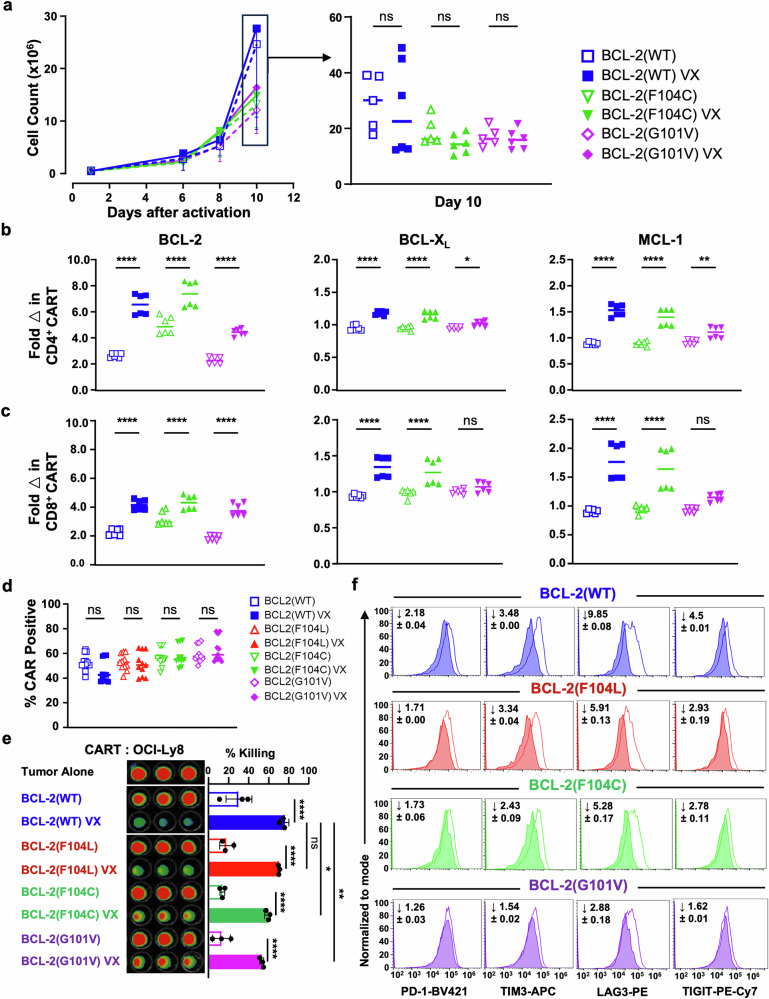
Venetoclax improves CART efficacy in a BCL-2-dependent manner. CART cells with various BCL-2 constructs treated with vehicle are indicated by an empty symbol, and venetoclax-treated CART (VX: 800 nM) are indicated by a filled symbol. **a** Proliferation curves of CART products at different time points. On right: Comparison of cell counts of each CD19CAR construct on day 10 of expansion demonstrates no significant changes in expansion between vehicle vs venetoclax-treated CART. **b**, **c** Anti-apoptotic BCL-2 family protein expression in different BCL-2 overexpressing CART constructs as measured flow cytometrically and normalized to the corresponding vehicle-treated CART by MFI fold change from products made from two healthy donors. BCL-2 expression was upregulated in all CART and further upregulated to different extents following treatment with venetoclax. CART with BCL-2 mutants shows decreased protein level changes of BCL-X_L_ and MCL-1, correlating to their relative levels of venetoclax resistance. **d** CAR expression is comparable among T cells transduced with different CAR constructs and remained similar whether treated with vehicle or venetoclax. **e** CART expressing BCL-2 mutants shows attenuation of venetoclax-induced antitumor activity compared to venetoclax-treated CART expressing BCL-2(WT). **f** Venetoclax-treated CART with BCL-2 mutants show loss of venetoclax-related ability to resist exhaustion. A fold decrease in expression of PD-1, TIM3, LAG3, and TIGIT between venetoclax- and vehicle-treated CART is indicated in each histogram. **a**–**c** Data are from CART prepared from two healthy donors with three technical replicates, and MFI normalized to the corresponding vehicle-treated donor’s CART. **d** Data summarized from three independent experiments with three healthy donor samples and three technical replicates each. **e**, **f** Representative data from one experiment out of three independent experiments. **a**–**d** Data shown as mean ± SD. Two-way ANOVA with SIDAK’s multiple comparison tests was performed. ^*^P < 0.05, ^**^P < 0.01, ^****^P < 0.0001, ns non-significant

To evaluate whether the effects following venetoclax treatment in CART cells were due to on-target drugging of BCL-2, we designed CART vectors that overexpressed genes encoding either wild-type BCL-2 (BCL-2(WT)) or one of three BCL-2 constructs with mutations within the BH3 binding pocket known to incur various levels of venetoclax resistance.^37–39^ Of these mutations, BCL-2(F104L) has the least resistance to venetoclax binding compared to BCL-2(F104C) and BCL-2(G101V) mutations.^39^ We hypothesized that if our observations were specific to BCL-2 drugging, then attenuation of these effects would correlate with venetoclax resistance. We focused first on examining CART constructs with the most resistant forms of BCL-2 and compared them to CART overexpressing BCL-2(WT). There were no significant differences in the final product cell counts between vehicle- and venetoclax-treated CART, although CART with BCL-2(WT) showed a trend towards greater proliferation (Fig. 3a). BCL-2 protein levels were increased in all CART cells regardless of the BCL-2 isoform overexpressed and was uniformly increased following venetoclax treatment, as measured previously (Fig. 3b, c). Notably, however, the fold increase in other anti-apoptotic proteins was greatest in venetoclax-treated CART BCL-2(WT) compared to CART cells containing mutant isoforms of BCL-2. These data suggest that binding of venetoclax to the BCL-2 BH3 pocket is critical for the observed increases in BCL-X_L_ and MCL-1. Next, to ensure equivalent comparisons of therapeutic efficacy, we measured no differences in CAR expression between the CART populations (Fig. 3d). In further support of venetoclax binding to BCL-2 as the on-target mechanism responsible for increased CART-mediated activity, tumor cell killing was highest by venetoclax-treated CART BCL-2(WT) and lowest by CART BCL-2(G101V) (Fig. 3e). Thereby, while venetoclax was still able to target endogenous wild-type BCL-2 within all CART constructs, killing was significantly attenuated in mutant BCL-2-containing CART cells. Furthermore, CART cells co-expressing mutant forms of BCL-2 regained an exhaustion phenotype similar to vehicle-treated cells following incubation with tumor cells (Fig. 3f). This finding was directly proportional to the relative affinities of BCL-2 for venetoclax. Collectively, these results confirm that the effects of venetoclax on CART are due to direct binding to the BCL-2 BH3-binding pocket. Moreover, these changes are due to alterations beyond apoptotic modulation and suggest alterations in other T cell signaling pathways.

### Venetoclax induces universal transcriptional changes in CART

**Fig. 4:**
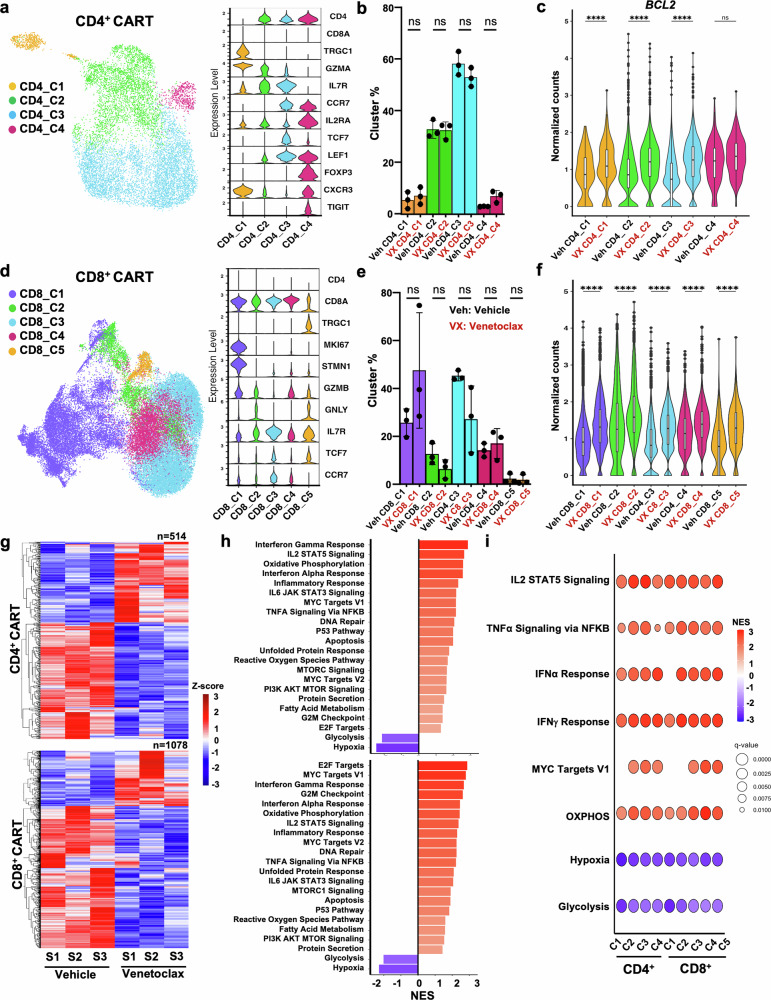
Gene expression analysis of venetoclax-treated CART reveals transcriptional T cell reprogramming. Vehicle or venetoclax-treated CART were generated as in Fig.1a, sorted for live cells, and analyzed by scRNAseq on day 13 after expansion. The experiment was performed using three biological replicates. **a**, **d** Uniform manifold approximation and projection (UMAP) embedding of clusters in CD4^+^ and CD8^+^ CART by scRNAseq. Key genes used for cluster designation are indicated. **b**, **e** Venetoclax-treated CART show similar cluster proportions compared to vehicle-treated cells. The proportion of each cluster within its respective treatment group are indicated by dots representing individual donors. **c**, **f** *BCL2* transcript level compared between each cluster of CD4^+^ and CD8^+^ CART treated with vehicle or venetoclax shows that venetoclax upregulated *BCL2* globally in all cell clusters. **g** Venetoclax-treatment led to a distinctive alteration in CART transcriptional profiles, as indicated by a heatmap of pseudo-bulk *Z*-score-scaled expression of DEGs with BH-adjusted *P* value < 0.05, avg_log2FC > 0.58. **h** Highly upregulated and downregulated pathways in venetoclax-treated CART compared to vehicle-treated CART. **i** HALLMARK pathway analysis across CART cell subpopulation clusters demonstrates global alterations. **b**, **c**, **e**, **f** Data shown as mean ± SD. Two-way ANOVA with SIDAK’s multiple comparison tests was performed. ^****^P < 0.0001, ns non-significant. Veh: Vehicle (black) and VX: venetoclax (red) in parts **b**, **c**, **e**, **f**

Based on the above results, it appeared that the presence of BCL-2 and not alterations in the CD4^+^/CD8^+^ T cell ratio or memory phenotype was directly associated with the increased efficacy of venetoclax-treated CART cells. Therefore, we hypothesized that venetoclax induced gene expression reprogramming leading to enhanced T cell signaling and effector-like characteristics. To test this, we first performed bulk RNAseq on sorted CD4^+^ CAR^+^ and CD8^+^ CAR^+^ T cells prepared from PBMCs from three healthy donors following either vehicle or venetoclax treatment. Venetoclax led to distinct transcriptional profiles in both CD4^+^ and CD8^+^ CART cells as measured by principal component analysis (PCA) (Supplementary Fig. S4a) and resulted in alterations in genes representing similar signaling pathways within each cell type as determined by HALLMARK analysis (Supplementary Fig. S4b). We next sought to identify whether these gene expression changes were specific to certain CART cell subpopulations. In this context, we performed single-cell analysis (scRNAseq) on vehicle- or venetoclax-treated CART prepared from an additional three healthy donors to measure effects of venetoclax on CD4^+^ and CD8^+^ CART subsets (Fig. 4a, d). Venetoclax treatment did not significantly alter the cluster designation in either the CD4^+^ or CD8^+^ CART products (Fig. 4b, e). However, while there were no significant differences in the relative percentage of individual clusters between venetoclax- and vehicle-treated CART, there was a trend for venetoclax-treated cells to have signatures reflective of increased “proliferative” (cluster 1; e.g., MKI67) and less “naïve” (cluster 3; e.g., CCR7/TCF7/IL7R) states within CD8^+^ CART cells (Fig. 4b, e). Notably, *BCL2* was significantly increased across all venetoclax-treated CD4^+^ and CD8^+^ CART cell clusters relative to vehicle-treated cells (Fig. 4c, f). scRNAseq identified 514 and 1078 differentially expressed genes (DEGs) in CD8^+^ and CD4^+^ T cells, respectively (Fig. 4g). Using gene set enrichment analysis (GSEA) against HALLMARK pathways, we found that venetoclax-treatment promoted the enrichment of similar pathways between CD4^+^ and CD8^+^ CART, suggesting a shared mechanism of action (Fig. 4h). Many of the significantly altered transcription programs were again associated with T cell activation, effector function, cell cycle, survival and metabolism highlighted by positive enrichment of genes associated with IL-2/STAT5, PI3K/AKT/MTOR, MTORC1, MYC, and TNFα/NFκB signaling (Fig. 4h). Significant enrichment of these pathways was consistent across CD4^+^ and CD8^+^ CART cell clusters (Fig. 4i). GSEA also demonstrated upregulation of transcriptomic signatures associated with IFN-α, IFN-γ and TNF-α signaling (Supplementary Fig. S4c). To correlate alterations in BCL-2 family protein levels (Fig. 1c–e) to transcriptional changes, we found several BCL-2 family transcript changes, including upregulation of *BCL2, BCL2L1, BAX*, and *PMAIP1* (NOXA) and downregulation of *MCL1* and *BCL2L11* (BIM) in venetoclax-treated CART cells (Supplementary Fig. S4d). The upregulation of *BCL2* and downregulation of *BCL2L11* correspond with our previous work showing similar results in murine T cells treated with venetoclax.^24,25^ Low gene expression in the setting of MCL-1 protein increase (Fig. 1c–e) is a well described phenomena secondary to extension of its protein half-life following binding within its BH3 pocket, either following treatment with MCL-1-specific BH3 mimetics or through dissociation of BCL-2 family proteins from other anti-apoptotic family members and subsequent binding to MCL-1.^40,41^

Given the common enriched pathways shared between scRNAseq and bulk RNAseq signatures of CD4^+^ and CD8^+^ CART populations, we sought to identify whether select genes were core contributors to these enrichments. To do this, we identified genes that were most frequently present in the leading edge of each enrichment across both sets of data among all six donors. We found that most genes were shared across datasets, suggesting that core transcriptomic mechanisms existed across conserved pathways following venetoclax treatment (Supplementary Fig. S5). Altogether, these data indicate that the enhanced effect observed with venetoclax in CART results from broad-spectrum transcriptional reprogramming of T cells that modulate T cell signaling, functionality, and metabolic fitness of the CART product.

### The enhanced antitumor effect of venetoclax-treated CART is mediated in part by PI3K/AKT and JAK/STAT signaling

**Fig. 5:**
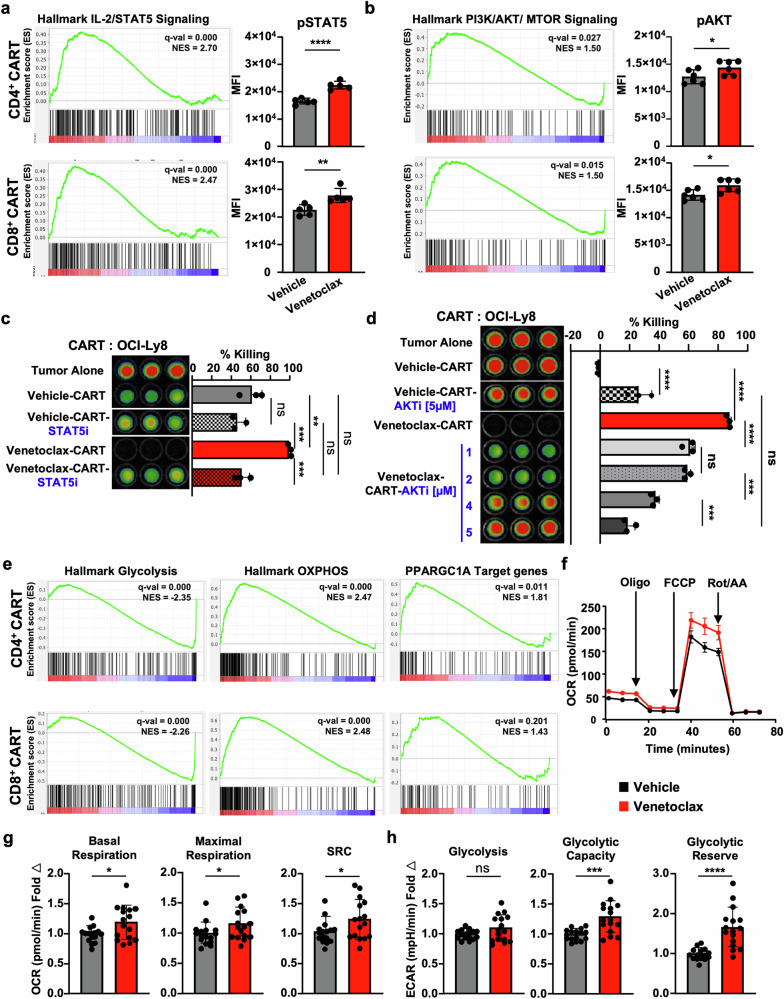
Venetoclax alters CART cell signaling and improves metabolic fitness. **a, b** Venetoclax-treatment results in transcriptional changes associated with IL-2/STAT5 and PI3K/AKT/MTOR signaling as indicated by GSEA of CD4^+^ and CD8^+^ CART scRNAseq. These changes are reflected in increased intracellular protein expression of (**a**) phospho-STAT5 (Tyr694) and **b** phospho-AKT (Ser473) in venetoclax-treated compared to vehicle-treated CART. Therapeutic inhibition of (**c**) STAT5 and (**d**) AKT reverses the increased killing effect following venetoclax treatment as measured using in vitro tumor killing assays against OCI-Ly8 DLBCL cells. Effector to tumor (E:T) ratio of 1:5 and incubation for 48 h prior to imaging. **e** Venetoclax-treatment results in transcriptional changes associated with decreased glycolysis and increased OXPHOS and *PPARGC1A*-related signaling as indicated by GSEA of CD4^+^ and CD8^+^ CART scRNAseq. **f** Venetoclax-treated CART cells have higher OXPHOS as measured by increased oxygen consumption rate (OCR). Venetoclax-treated CART also have increased (**g**) basal respiration, maximal respiration, and spare respiratory capacity (SRC) while still maintaining (**h**) higher glycolytic capacity and reserve. Seahorse data represent analysis of *n* = 8 technical replicates from two healthy donors of two independent experiments in mean ± SD (**g**, **h**) and mean ± SEM (**f**). Unpaired Student’s *t*-test was performed for (**a**, **b**, **g**, **h**), and one-way ANOVA with post-hoc Tukey’s tests was performed for (**c**, **d**)

Recent data suggest that PI3K/AKT and JAK/STAT5 signaling are key determinants of CART fitness, as defined by increased proliferation, effector function, memory, and survival.^30,42–44^ We previously reported that homeostatically expanding naïve murine CD4^+^ and CD8^+^ T cells acquire effector-like gene signatures following in vivo administration of venetoclax, marked by upregulation of PI3K/AKT and JAK/STAT signaling pathways.^24^ Aligned with this data, GSEA of scRNAseq of venetoclax-treated CART revealed a significant enrichment of genes associated with PI3K/AKT/MTOR and IL-2/STAT5 signaling (Fig. 5a, b). To confirm increased signaling within these pathways, CART cells were evaluated for the active phosphorylated forms of STAT5 and AKT. Western blot analysis showed that total CART expanded in the presence of venetoclax had increased pSTAT5 and pAKT when compared to vehicle-treated CART (Supplementary Fig. S6b, part I). This was also confirmed flow cytometrically in individual CD4^+^ and CD8^+^ CART cells (Fig. 5a, b). We then used pharmacologic inhibitors of STAT5 and AKT to evaluate if these pathways were responsible for the increased efficacy of venetoclax-treated CART products. Here, CART were expanded as detailed in Fig. 1a and then treated with either AC-4-130 (STAT5 inhibitor) or AKT inhibitor VIII (AKT 1/2 inhibitor) for 24 h prior to incubation with OCI-Ly8 cells.^45,46^ STAT5 inhibition significantly reduced venetoclax-treated CART-mediated killing to a greater extent than vehicle-treated CART (Fig. 5c and Supplementary Fig. S6a). We confirmed that STAT5 inhibition decreased pSTAT5 and pAKT in these cells (Supplementary Fig. S6b, part II). AKT inhibition also reduced the efficacy of venetoclax-treated CART (Fig. 5d). However, in contrast to STAT5 inhibition, and aligned with previously published reports, AKT inhibition resulted in increased efficacy of vehicle-treated CART (Fig. 5d).^47,48^ Assessment of these CART products indicated a greater decrease in pAKT in venetoclax-treated CART compared to vehicle-treated CART (Supplementary Fig. S6b, part III). Neither treatment resulted in changes in BCL-2 protein levels. Collectively, these results indicate a reliance on both STAT5 and AKT pathways for venetoclax-mediated effects on CART cell killing.

### Venetoclax promotes CART metabolic fitness

HALLMARK analyses of venetoclax-treated CART also showed positive enrichment of pathways associated with fatty acid metabolism, OXPHOS, and ROS and negative enrichment of signatures associated with glycolysis and hypoxia in both CD4^+^ and CD8^+^ CART, as well as distinct enrichment signatures in *PPARGC1A* (Figs. 4h and 5e). *PPARGC1A* encodes for PGC1α, a key metabolic regulator that promotes mitochondrial biogenesis, OXPHOS, and fatty acid β-oxidation, and results in enhanced T cell fitness, memory formation, and T cell-mediated antitumor potency.^49^ Therefore, we sought to determine if there was a connection between these transcriptional alterations and actual metabolic profiles of venetoclax-treated CART. To test this, the oxygen consumption rate (OCR) of vehicle- and venetoclax-treated CART following expansion was measured using Seahorse Mito Stress analyses.^50^ Venetoclax significantly increased basal OCR, maximal respiration, and spare respiratory capacity (SRC) in CART (Fig. 5f, g).

OCR is considered a primary source of ROS in T cells.^51,52^ Related to this, venetoclax has been found to enhance the antileukemic effector activity of patient-derived T cells through increased ROS due to impaired respiratory chain supercomplex formation. In that setting, increased T cell-mediated killing was inhibited by the antioxidant *N*-acetyl-L-cysteine (LNAC).^22^ Thus, we wondered if the killing capacity of venetoclax-treated CART depended on upregulated ROS production that could be linked to enhanced OXPHOS levels. To test this, we incubated venetoclax-treated CART with escalating doses of LNAC and found no effect on their tumor killing capacity (Supplementary Fig. S6c). Thereby, ROS induction does not appear to be involved in the effect of venetoclax on CART in our system.

We next measured the extracellular acidification rate (ECAR) of venetoclax-treated CART to determine if their glycolytic status differed from vehicle-treated CART. While glycolysis was not affected at baseline, venetoclax-treated CART had significantly elevated glycolytic capacity and glycolytic reserve compared to vehicle-treated cells (Fig. 5h). Additionally, transcriptional data revealed that venetoclax treatment upregulated key genes involved in OXPHOS and fatty acid metabolism (e.g., *CPT1A* and *FABP5)* and downregulated genes associated with glycolysis (e.g., *PDK1*, *PGK1*, *SLC16A3*, and *VEGFA)*, a pattern well known to increase CART persistence (Supplementary Fig. S7).^53^

Balancing of STAT5 and AKT signaling has been associated with differential metabolic effects on CART and effector T cells.^47,48,54–56^ To determine if STAT5 or AKT pathways were responsible for the metabolic changes in venetoclax-treated CART, Seahorse analyses were performed on CART in the setting of STAT5 or AKT inhibition (Supplementary Fig. S6d). In contrast to similar globally decreased metabolic effects following AKT inhibition in vehicle- and venetoclax-treated CART, as previously reported,^47^ STAT5 inhibition led to significant decreases in OXPHOS and glycolysis-related measurements only in venetoclax-treated CART, indicating that STAT5 signaling is primarily responsible for the observed venetoclax-mediated metabolic effects.

### Venetoclax-treated CART prepared from healthy donors has increased therapeutic activity against leukemia and lymphoma in vivo

**Fig. 6:**
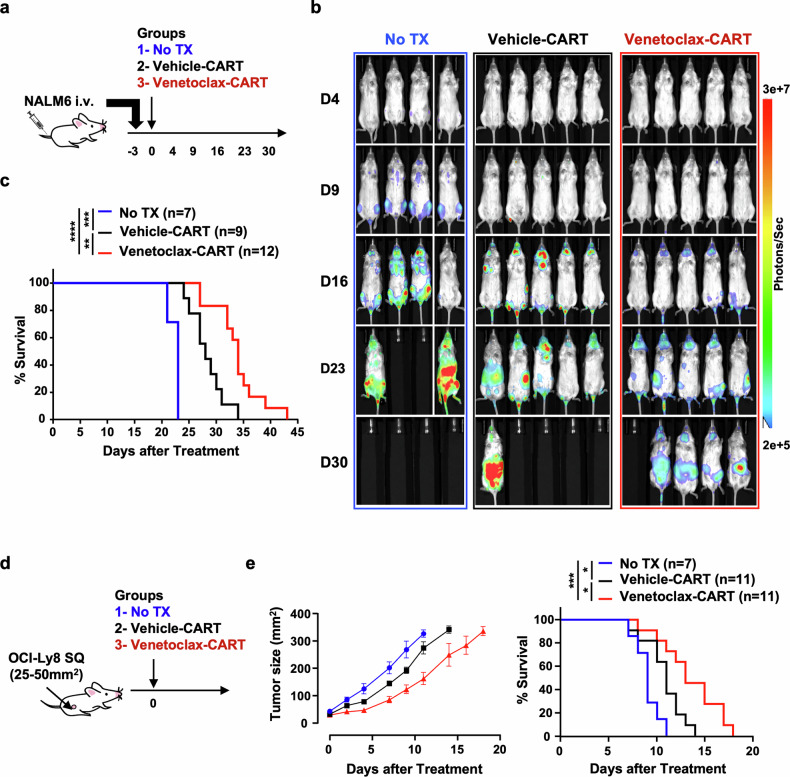
Venetoclax augments the in vivo efficacy of CART prepared from healthy donors. **a** Treatment schema for the NALM6 leukemia xenograft model. Three days following NALM6 injection, mice were randomly divided into groups that either received no treatment (No TX), vehicle-treated CART, or venetoclax-treated CART. **b**, **c** Animals that received venetoclax-treated CART had improved tumor control, resulting in significantly prolonged survival. **d** Treatment schema for the OCI-Ly8 DLBCL xenograft model. Mice with established subcutaneous (SQ) tumors (25–50 mm^2^) received no treatment (No TX), vehicle-treated CART, or venetoclax-treated CART. **e** Animals that received venetoclax-treated CART had improved tumor control and significantly prolonged survival. These results are pooled from three independent experiments. Data shown as mean ± SD. ^*^P < 0.05, ^**^P < 0.01, ^***^P < 0.001, ^****^P < 0.0001

Given the measured improvement of venetoclax-treated CART in vitro, we next sought to investigate whether these effects would translate in vivo. To measure this, two CD19^+^ xenograft tumor models were used, disseminated NALM6 leukemia and subcutaneously injected OCI-Ly8 DLBCL (Fig. 6a, d). In both cases, mice that received venetoclax-treated CART had delayed tumor growth (Fig. 6b, e) that translated into significantly improved survival (Fig. 6c, e). Vehicle- and venetoclax-treated CART could be detected in animals prior to reaching advanced stages of disease. There were greater ratios of T_EM_ CD8^+^ CART following tumor challenge in those animals that received venetoclax-treated CART compared to more terminally differentiated (T_EMRA_) CART in animals that received vehicle-treated CART (Supplementary Fig. S8)

### Venetoclax amplifies the therapeutic potency of CART prepared from patient-derived T cells

**Fig. 7:**
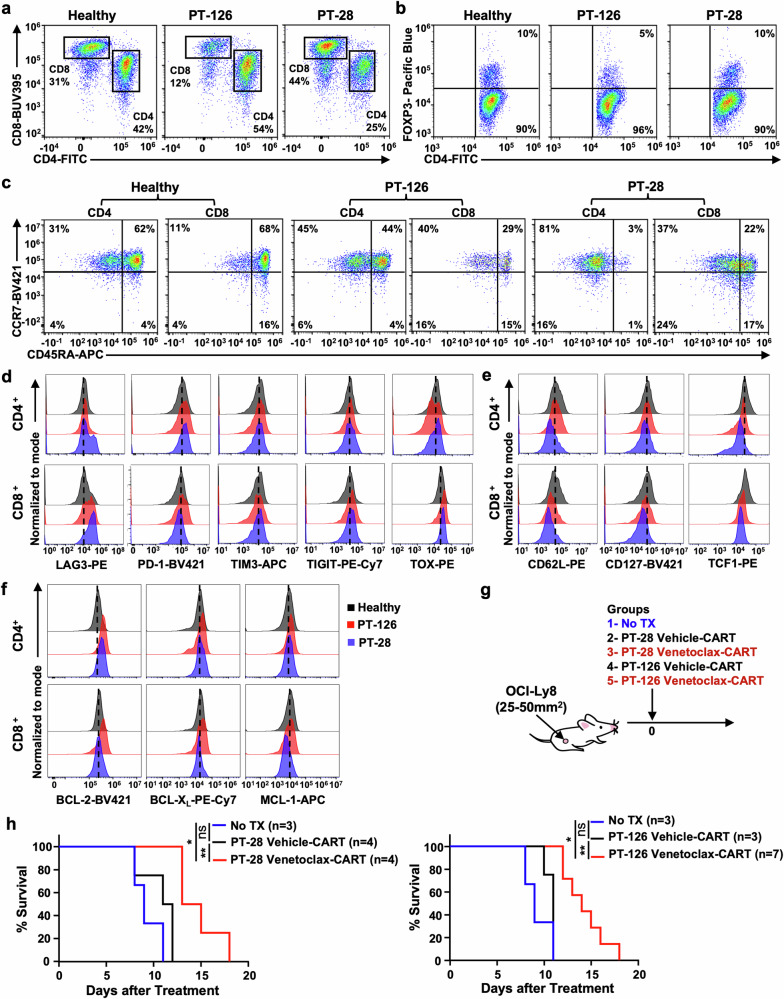
Venetoclax enhances the potency of CART prepared from patient samples regardless of their starting T cell composition, memory phenotype, or exhaustion profiles. Patient-derived PBMCs (PT-28 and PT-126) collected at the time of commercial CART cell apheresis were used to isolate T cells and prepare vehicle or venetoclax-treated CART. PBMCs from healthy volunteers were used as comparators. Starting material was evaluated for (**a**) CD4:CD8 ratio, **b** CD4^+^ FOXP3^+^ Treg content, **c** CD4^+^ and CD8^+^ T cell subpopulation content as determined by CCR7 and CD45RA, **d** exhaustion phenotype based on surface expression of LAG3, PD-1, TIM3, TIGIT and intracellular marker TOX, **e** T cells memory status based on the surface expression of CD62L and CD127 and intracellular expression of TCF-1, and **f** protein expression levels of BCL-2, BCL-X_L_, and MCL-1. **g** Mice with established subcutaneous OCI-Ly8 tumors (25–50 mm^2^) received no treatment (No TX), vehicle-treated CART, or venetoclax-treated CART as indicated. **h** Treatment with venetoclax-treated CART prepared from patient samples (PT-28 or PT-126) resulted in prolonged survival compared to vehicle-treated CART. The number of mice in each group is given. Data shown as mean ± SD. ^*^P < 0.05, ^**^P < 0.01, ns non-significant

We next tested if venetoclax could enhance the efficacy of CART prepared from chemotherapeutically pretreated patient-derived T cells that were isolated at the time of apheresis for commercial CD19^+^ CART products (Supplementary Table S1). Starting material from each sample was flow cytometrically characterized for its CD4:CD8 ratio, Treg content, memory phenotype, exhaustion markers, and anti-apoptotic protein expression. CD4:CD8 ratios between samples were markedly different, while the percentages of FOXP3^+^ CD4^+^ Tregs were generally similar between samples (Fig. 7a, b and Supplementary Fig. S9a, b). The relative percentages of naive (CCR7^+^, CD45RA^+^), central memory (CCR7^+^, CD45RA^−^), effector memory (CCR7^−^, CD45RA^−^), and effector memory RA^+^ (TEMRA, CCR7^−^, CD45RA^+^) T cells across samples showed that patient samples universally had fewer naïve T cells compared to those from healthy volunteers (Fig. 7c and Supplementary Fig. S9c). Furthermore, patient-derived CD4^+^ and CD8^+^ T cells had increased expression of T cell exhaustion markers, particularly LAG3 and PD-1, compared to those from healthy donors, and had less memory characteristics reflected by decreased levels of CD62L, CD127, and TCF1 (Fig. 7d, e and Supplementary Fig. S9d). There were also heterogeneous baseline protein levels of BCL-2, BCL-X_L_, and MCL-1 between the samples (Fig. 7f and Supplementary Fig. S9e). BCL-2 protein levels were particularly different across samples, which may be pertinent given the recent finding that BCL-2 expression in T cells from patient apheresis products positively correlated with CART response.^28^ While there were differences in overall CAR transduction efficiency between samples, there were no significant differences in CAR expression between vehicle- and venetoclax-treated samples within individual subjects (Supplementary Figs. S9f and S10a). There were, however, marked differences between the final CART cell CD4:CD8 ratios between donors, which were somewhat reflective of their starting T cell ratios (Fig. 7a and Supplementary Fig. S10b). Not surprisingly, there was a greater percentage of CAR^+^ FOXP3^+^ Tregs in CART products manufactured from T cells collected from patients compared to healthy donors. Venetoclax treatment did not affect these percentages (Supplementary Fig. S10c). Despite differences in starting material and post-production CD4^+^:CD8^+^ CART cell ratios, venetoclax treatment significantly enhanced the in vitro and in vivo CART efficacy of all patient-derived CART products (Fig. 7g, h and Supplementary Fig. S9g). These results indicate that venetoclax treatment during ex vivo expansion can reprogram CART cells and increase their efficacy irrespective of the starting T cell material. These results warrant further investigation into the use of venetoclax to reprogram patient-derived T cells for use in CART-related and other adoptive T cell treatments.

## Discussion

In this study, we functionally and mechanistically reveal how therapeutically targeting BCL-2 in T cells with venetoclax, a BCL-2-specific BH3 mimetic, can reprogram the fitness and therapeutic efficacy of CART cell products. While a vast body of literature describes the clinical use, apoptotic mechanisms of action, and therapeutic resistance of venetoclax in cancers, less is known about how venetoclax affects non-malignant immune cells. Our results support the notion that anti-apoptotic BCL-2 proteins represent a new regulatory node that can be therapeutically targeted to modulate T cell function.

A growing body of recent literature has found that venetoclax alters the immune function of natural killer cells, dendritic cells, and Tregs.^57,58^ In the current study, we sought to understand the impact of venetoclax on human T cell function using CD19CART as a model system. We found that BCL-2 drugging during CART production induces an adaptive-reprograming that protects CART cells from apoptosis while at the same time amplifies their cytotoxic effects via activation of specific T signaling pathways. This adaptive reprogramming is marked by increased protein expression of BCL-2 in addition to other key anti-apoptotic proteins known to play various survival roles in T cells, such as BCL-X_L_ and MCL-1 (Fig.1c–e). This protein upregulation appears to occur through both transcriptional activation, particularly of *BCL2* and *BCL2L1* (BCL-X_L_) (Supplementary Fig. S4d), and via protein stabilization through well-described mechanisms of dissociation of pro-apoptotic BAX/BAK and BH3-only proteins (e.g., BIM) and subsequent sequestration by other anti-apoptotic proteins, such as MCL-1, which prevents proteosome-mediated degradation.^41^ Upregulation of BCL-X_L_ and MCL-1 in venetoclax-resistant tumors has also been correlated to the upstream activation of NFκB and STAT5 pathways, as well as crosstalk between JAK/STAT5 and PI3K/AKT.^59–61^ Our data confirm enrichment of these same pathways in venetoclax-treated CART cells and that their inhibition decreases venetoclax-related effector CART function (Figs. 4h, i and 5a–d).

It is well-known that STAT5 signaling is critical for the maintenance of effector and memory function of T cells.^62,63^ Thus, genetically targeting STAT5 has been an appealing strategy to improve the efficacy of adoptively transferred T cells. Constitutive expression of STAT5 in CD4^+^ T cells can lead to activation of epigenetic and transcription reprogramming, enabling T cells to resist exhaustion, promote in vivo persistence, and enhance polyfunctionality, all leading to increased antitumor efficacy.^30^ Other recent work has discovered that STAT5 activation is able to antagonize TOX-induced epigenetic-exhaustion programming to functionally rewire exhausted CD8^+^ T cells towards an effector-like state.^64^ Coincidentally, BCL-2 protein expression has been reported to be a downstream requirement for STAT5 to maintain the survival of T cells in effector and memory-like states.^30,63,64^ Our data suggests, that BCL-2 or its binding partners, could serve as a targetable upstream regulator of STAT5, thus pointing to a BCL-2/STAT5 axis as a possible driver for the recently reported enhanced antitumor efficacy accompanied by overexpressing BCL-2 in CART.^27,28^

In addition to STAT5, we also found that the AKT pathway was activated following BCL-2 blockade during CART production. While activation of the PI3K/AKT/mTOR pathway has been reported to induce T cell effector activity in terminally differentiated T cells, this activation ultimately decreases their antitumor efficacy. Therefore, inhibition of PI3K and AKT in CART has been explored to generate a greater memory T cell phenotype with superior antitumor killing.^42,47,48,65–67^ However, AKT inhibition diminished, rather than amplified, venetoclax-induced antitumor efficacy of CART in our model (Fig. 5d). Thus, it appears that venetoclax induces a unique interdependence on AKT and STAT5 signaling in CART cells. There is precedence for this, as other studies have shown the importance of STAT5 to activate, sustain, and allow low-level activation of PI3K/AKT to accumulate in cells, which then sequentially regulates the metabolic needs of T cells at rest and following stimulation.^54,56,68^ We hope that further investigation following venetoclax treatment in CART cells will shed light on how these signaling pathways are reprogrammed to alter effector and metabolic pathways.

Metabolic parameters of CART cells were also altered by venetoclax. Effector T cells are characterized by having increased glycolysis used to meet the metabolic demands for activation and cytokine production. However, maintaining low metabolic activity during CART manufacturing has been used to favor the production of less differentiated, more memory-like cells with improved in vivo persistence.^69,70^ Several studies have shown that T_CM_ cells display higher basal OCR and SRC compared to T_EM_ and T_EMRA_ through increased dependence on fatty acid oxidation.^50,53,71^ Further, several metabolic strategies that inhibit glycolysis, promote OXPHOS, or induce PGC1α have been reported to maintain T cells in a less differentiated state and thus enhance CART efficacy.^70,72,73^ Our results indicate that venetoclax may induce metabolic features that enhance the overall metabolic fitness of the CART product through promotion of a more memory-like rather than effector-like metabolic status in CART, while also enhancing their ability to meet the metabolic requirements for effector function following antigen stimulation.

Importantly, we validated that the measured effects of venetoclax were due to on-target BCL-2-dependent drugging. CART constructs overexpressing BCL-2 mutants with decreased binding to venetoclax had a reduction in protein levels of BCL-X_L_ and MCL-1 (Fig. 3). Presumably, venetoclax caused a rewiring of the anti-apoptotic family through dissociation of BCL-2 binding partners and stabilization of BCL-X_L_ and MCL-1. BCL-2 point mutation-mediated venetoclax resistance also correlated with decreased CART cell function, supporting the notion that direct binding to BCL-2 by venetoclax is responsible for venetoclax-related effects. In these experiments, it is important to point out that endogenous BCL-2 was still present in these treated CART cells and that binding of endogenous BCL-2 family proteins to these mutant isoforms is unchanged.^39^ This was likely responsible for the baseline increased tumor cell killing and relatively small, yet significant (~10% incremental decrease) changes following venetoclax treatment compared to vehicle-treated cells in these CART. We believe that dynamic changes within the BCL-2 family following venetoclax treatment may be contributing to the signaling and metabolic changes in CART and warrant further investigation.^74^

Dysfunctional, exhausted, and terminally differentiated T cells from patients with malignancies and pretreated with repetitive cycles of chemotherapy result in poor CART products that often lead to tumor relapse.^75–78^ Our data shows that venetoclax treatment of CART manufactured from T cells isolated from patients enhances their antitumor efficacy regardless of the starting material’s exhaustion phenotype, memory status, or CD4^+^/CD8^+^ T cell content. This data supports other work showing an increase in anti-tumor T cell-mediated killing in patients with AML who are treated with venetoclax. This effect is via unclear mechanisms but appears to be associated with the induction of T cell populations with high BCL-2 expression and progenitor/non-exhausted effector-like properties.^22,23^ Together, these findings support our results showing that venetoclax treatment of patient-derived T cells could be used to overcome inherent T cell insufficiencies that would otherwise lead to the poor antitumor therapeutic outcomes of adoptive T cell therapies, like CART. However, additional clinical studies are needed to broadly test this hypothesis in patients.

One caveat to our study is that venetoclax treatment was ex vivo and not in vivo. We did this to ensure that the measured effects were from direct targeting of BCL-2 in T cells. This raises the question, however, if continued BCL-2 blockade is needed to maximize clinical benefit. Recent studies have raised concerns about combining in vivo administration of venetoclax with CART therapy because of the potential apoptosis of CART cells. To bypass this, CART overexpressing BCL-2(F104L) has been tested in vivo with combination venetoclax to prime tumor cells for apoptosis while simultaneously protecting CART cells from cell death.^28^ Another recent study found that CART overexpressing BCL-2(G101V) or BCL- X_L_(WT) had increased tumor cell killing in vivo in the presence or the absence of venetoclax.^27^ This suggests to us that BCL-2 or BCL-X_L_ have inherent immunomodulatory capabilities beyond increasing cell survival. The conceptual difference between these studies and ours is that we applied BCL-2 therapeutic “pressure” during ex vivo expansion and thereby forced apoptotic and functional adaptation in the CART product prior to infusion, thus proving a direct immunomodulatory effect of venetoclax.

One limitation of our results is that the venetoclax-mediated effects on CART function and tumor eradication do not appear long-lived in vivo. This could be attributed to a number of reasons including: (1) the effect is transient and needs to be continued with in vivo therapeutic BCL-2 family drugging, (2) CART cells that encounter tumor targets proliferate and thus have “undrugged” nascent BCL-2 production, (3) venetoclax-treated CART become dysfunctional through a novel exhaustion reprogram, or (4) the use of immunocompromised animals in xenograft models limit host immune interactions that may provide additional immunological help in eradicating tumors.

In summary, our study provides novel insights regarding immunologic effects orchestrated by BCL-2 modulation, which can be leveraged through the therapeutic use of venetoclax in expanding CART cells. We believe that these results further support the clinical testing of BH3 mimetics as immune modulatory compounds.

## Materials and methods

### Cell lines and general cell culture

A20 murine B cell lymphoma tumor was purchased through ATCC. Human OCI-Ly8.Luci.Chili, OCI-Ly8.CD19KO.Luci.Chili, NALM6.Luci.Chili was generously provided by Dr. Jun Huang (University of Chicago). OCI-Ly8.CD19KO.Luci.Chili was generously provided by Dr. Justin Kline (University of Chicago). Cells were cultured at a concentration of 1 × 10^6^ cells/mL of standard culture media (RPMI 1640 (Gibco) + 10% fetal bovine serum albumin (FBS), 1% penicillin/streptomycin, 1% HEPES, 1% GlutaMAX, 1% Sodium pyruvate, 1% non-essential amino acids, and 0.1 µM 2-mercaptoethanol at 37 °C in 5% CO_2_ incubator. All cell lines were tested for Mycoplasma contamination (Lonza).

### Virus production and transduction of CAR-engineered murine and human T cells

The retroviral vector MSGV-1D3-28Z.1–3 containing mouse CD19-targeting CAR (mCD19CAR) was generously provided by James N. Kochenderfer (National Cancer Institute).^31^ Retroviral supernatants preparation and murine T cell retroviral transduction procedures were described previously.^30^ The Lentiviral vector SFG-humCD19_4-1BB containing human CD19-targeting CAR (hCD19CAR) was generously provided by Dr. Jun Huang (University of Chicago). Replication-defective, second-generation lentiviral vectors were produced using Lenti-X 293 T cells (Takara Bio). Approximately 5 × 10^6^ to 7 × 10^6^ cells were plated in T75 in standard culture media (DMEM + 10%FBS, without antibiotics) and incubated for 48 h at 37 °C. Then, cells were transfected with hCD19CAR lentiviral vector using a combination of Polyethylenimine (PEI) (Polysciences #23966-2) (64 μL, Invitrogen); pMD2.G (2 μg), and pCMV-dR8.2-dvpr (6 μg) packaging plasmids; and 8 μg of expression plasmid (CAR). PEI and plasmid DNA were diluted in 0.8 mL Opti-MEM (Thermo Scientific # 31985062) media prior to transfer into lentiviral production flasks. At both 24- and 48-h following transfection, culture media containing viral particles were harvested and concentrated using Lenti-X Concentrator (Takara Bio) following the manufacturer’s instructions. PBMC samples were either collected from healthy donor volunteers under University of Chicago Institutional Review Board-approved protocols, IRB#14-0221-CR009, or from patient apheresis samples provided by UChicago cellular therapy biobank, IRB #18-0025. Human T cells were isolated from PBMC using the Pan T Cell Isolation Kit following the manufacturer’s instructions (Miltenyi Biotec). Purified T cells were activated by T-Activator CD3/CD28 Dynabeads (ThermoFisher) at a ratio of 1:1 beads/cell in the presence of human IL-2 (200–500 U/mL) and incubated at 37 °C overnight. Activated T cells were spin-infected with hCD19CAR lentiviral supernatant using protamine sulfate (Sigma-Aldrich # P3369-10G) at a multiplicity of infection (MOI) = 5. Human T cells were further expanded for 10–13 days in the presence of IL-2 before harvesting for in vitro and in vivo studies.

### Designing CAR constructs overexpressing BCL-2

HiFi Assembly (NEB) was used to combine CAR and BCL2 transgenes into the second-generation lentiviral transfer plasmid pHR. The P2A ribosomal skip peptide was inserted between CAR and BCL2 sequences for bicistronic expression under the same promoter. Assembled products were transformed into STABLE chemically-competent *E*. *coli* (NEB) and plated on carbenicillin antibiotic plates. Individual colonies were inoculated and plasmid DNA isolated (Qiagen). Size verification was carried out by restriction enzyme digestion and gel electrophoresis. Sanger sequencing spanning at minimum the transgene promoter through the stop codon was used to select final colonies. Mutagenic primers were then used to introduce F104L, G101V, or F104C substitutions into the BCL2 gene via site-directed mutagenesis.

### Luminescence-based cytotoxicity and serial killing assay

Cytotoxicity was performed by a coculture of CART cells with luciferase-expressing tumor cells (OCI.Ly8 and Nalm6) in triplicate at E:T ratios (1:5) for a period of approximately 48 h. D-Luciferin was added before plate imaging by a Lago X imager, and tumor signal was quantified using Aura software v.4.0.7. The following equation was used for calculation of specific lysis percentage for cytotoxicity assays: % Killing = [(tumor cell only luminescence – luminescence of CART cell + tumor)/tumor cell only luminescence] × 100.

Serial killing assays were performed using a coculture of CART cells with luciferase-expressing tumor cells (OCI.Ly8) in triplicate at E:T ratios (1:1). Killing was monitored daily. Tumor cell signal was monitored at different time points and compared between the two tested conditions (vehicle vs venetoclax-treated CART). When the tumor signal disappeared, indicating tumor clearance (and confirmed using flow cytometry), CART cells were separately harvested, re-counted, and co-cultured with fresh OCI.Ly8 tumors at an E:T ratio of 1:1. This process was repeated through multiple rounds of tumor cell challenge.

### Flow cytometry assays

For surface marker staining, cells were stained with fluorophore-conjugated surface antibodies (Supplementary Table S2) for 15 min at room temperature, washed, and re-suspended in FACS buffer (PBS + 2% FBS) before analysis. BCL-2 protein flow analysis was performed as previously described.^79^ For intracellular staining, surface marker staining was initially performed, followed by fixation and permeabilization using the FOXP3 Transcription Factor Staining Buffer Set according to the manufacturer’s protocol (eBioscience). To detect pSTAT5 and pAKT in CART cells, cells were fixed in 2% formaldehyde, followed by permeabilization with ice-cold methanol. After washing with phosphate-buffered saline (PBS), cells were stained with pSTAT5 antibody at room temperature for an hour before washing and flow cytometry analysis. For intracellular cytokine detection, CART cells were cultured with OCI.Ly8 tumor cells at a 1:5 ratio for 1 h, then a mix of GolgiStop and GolgiPlug was added for an additional 3 h before harvesting cells for staining. Cells were harvested and surface-stained, followed by cytokine staining with an intracellular fixation and permeabilization kit (BD Biosciences). For cell exhaustion, CART cells were co-cultured with OCI.Ly8 tumor cells at a 1:3 ratio for 48 h, followed by surface staining for the indicated exhaustion markers. To monitor cellular apoptosis, fluorescent caspase-3/7 substrates for detecting apoptosis in intact cells; NucView® Caspase-3 Enzyme Substrates (Biotium #10405) were used following the manufacturer’s protocol. Data were acquired on a NovoCyte Penteon (Agilent). For bulk RNA sequencing, CAR^+^ T cells were sorted using a BD FACSAria Fusion. All data analyses were performed using FlowJo 10.8.1 software (FlowJo, LLC) or NovoExpress software (Agilent). All reagents are listed in Supplementary Table S2.

### Western blot analyses

Whole-cell protein lysates were obtained by preparing cells in RIPA buffer (Thermo Fisher Scientific) with 2.4 mM sodium orthovanadate and protease inhibitor cocktail. Proteins (20–25 ug) were loaded onto a 4–12% SDS-PAGE gel, size distributed, and transferred to a nitrocellulose membrane. Membranes were blocked with 5% milk in 1X TBST before overnight incubation at 4 °C with primary antibodies against p-AKT (S473; 1:500; Cell Signaling Technology, #4060), AKT (1:1000; Cell Signaling Technology, #9272), p-STAT5 (Y694; 1:500; Cell Signaling Technology, #9351), STAT5 (1:1000; Cell Signaling Technology, #25656), BCL-2 (1:1000; Cell Signaling Technology, #4223), BCL-XL (1:1000; Cell Signaling Technology, #2764), MCL-1 (1:1000; Cell Signaling Technology, #5453), and β-Actin (1:3000; Cell Signaling Technology, #3700). Primary antibodies were washed out with 1× TBST before secondary antibody incubation with anti-rabbit or anti-mouse IgG antibodies conjugated to HRP (product). Bands were quantified and normalized to β-Actin using ImageJ software. Uncropped Western blot images can be found in the supplementary material (Supplementary Fig. S11–S13).

### Cytokine secretion assays

CART killing assays were performed by co-culturing 0.1 × 10^6^ CART cells with 0.5 × 10^6^ tumor cells in 200 μL of complete T cell medium without IL-2 in a 96-well plate, all in triplicate. Forty-eight hours after co-culture, culture supernatants were collected, diluted 10- to 100-fold, and analyzed for IFNγ, TNFα, IL-2, and Granzyme B using ELISA LEGEND MAX kits (BioLegend). Absorbance readings were collected on the SpectraMax iD5 multi-mode microplate reader (Molecular Devices).

### TRAIL neutralization assay

CART cells were incubated for 30 min with various concentrations of TRAIL neutralizing antibody (anti-TRAIL, R&D Systems #MAB375) at twice the final solution concentration (100 μL) for 30 min at room temperature. Then, CART cells were co-cultured with OCI-Ly8.luciferase.chili cells at a 1:5 E:T ratio, with solution volume being diluted to the indicated final concentration in solution (200 μL). Co-culture plates were imaged on IVIS.

### Experimental mice, xenograft mouse model, in vivo treatment, and imaging

BALB/c mice on the CD45.1 background and immunodeficient non-obese diabetic (NOD)–severe combined immunodeficient (SCID) IL-2RƔ-null (NSG; NOD.Cg-*Prkdc*^*scid*^I*l2rg*^*tm1Wjl*^/SzJ) mice were purchased from Jackson Laboratory. Splenocytes from CD45.1 mice (6–10 weeks old) treated with vehicle or venetoclax 5 days per week for 3 weeks (total 15 doses) were harvested and used to prepare murine CART for in vitro studies. Venetoclax was reconstituted in 10% ethanol, 30% PEG 400, and 60% Phosal and was administered via oral gavage at a dose of 50 mg/kg.

To establish the OCI-Ly8 subcutaneous (SQ) xenograft mouse model, 5 × 10^6^ cells of OCI-Ly8 tumors were prepared in 100 μL of PBS containing 50% Matrigel (Corning) and implanted into the right flank of NSG mice (5–8 weeks old) via subcutaneous injection. The 3.5 × 10^6^ CAR^+^ cells were administered intravenously when the tumor size reached ∼30–50 mm^2^. Tumors were measured three times a week by caliper, and tumor size was calculated according to the equation: tumor size = (*L* × *W*), where L is the longest axis of the tumor, and *W* is the axis perpendicular to *L*. For the SQ tumor model, the experimental endpoint was achieved when the tumor size reached ~350 mm^2^. For the systemic intravenous (i.v.) xenograft mouse model, 1 × 10^6^ cells of NALM6.Luci.Chilli tumors in 200 μL of PBS were injected in tail vein of NSG mice. After three days, mice were either untreated or treated with vehicle or venetoclax pre-treated CART (1.5 × 10^6^ CAR^+^ cells). NALM6.Luci.Chilli engraftment and progression were monitored using Spectrum IVIS bioluminescence imager and quantified using Living Image software v.4.7.3 (Perkin Elmer), or by a Lago X imager and quantified using Aura software v.4.0.7 (Spectral Instruments Imaging), after mice were injected intraperitoneally (i.p.) with 150 mg/kg D-Luciferin (PerkinElmer # 122799). Imaging was performed under isoflurane anesthesia. For this systemic tumor model, animals were monitored for signs of disease progression and endpoints including >15% loss in body weight, fur loss, diarrhea, and disease-related hind limb paralysis. All mice were housed under specific pathogen-free conditions. All animals and experiments were approved by and performed in accordance with the guidelines and regulations set forth by the Institutional Animal Care and Use Committee of the University of Chicago.

### Seahorse assays

Seahorse Bioscience Analyzer XFe96 (Agilent) was used to perform metabolic analyses. Briefly, 0.2 × 10^6^ cells were resuspended in Seahorse XF DMEM Medium supplemented with 10 mM glucose, 1 mM sodium pyruvate, 2 mM glutamine, and plated on a Cell-Tak (Corning #354240)-coated microplate, allowing the adhesion of CART cells. Mitochondrial activities were measured by the OCR (pmol/min) using Seahorse Mito Stress Test kit (Agilent # 103015-100) with the real-time injections of oligomycin (2 μM), carbonyl cyanide 4-(trifluoromethoxy) phenylhydrazone (FCCP; 1 μM), and rotenone and antimycin (Rot/AA 0.5 μM). Glycolytic parameters were evaluated by measuring ECAR (mpH/min) using the Glycolysis Stress Test Kit (Agilent #103020-100) with the addition of glucose (10 mM), oligomycin (2 μM), and 2-deoxy-glucose (2-DG; 50 mM). Respiratory parameters were acquired and calculated according to the manufacturer’s instructions (Seahorse Bioscience). Reagents are listed in Supplementary Table 2.

### scRNAseq

Single-cell suspensions of vehicle- or venetoclax-treated CART of three healthy donors were prepared and stained with Zombie NIR Dye to sort live cells. Sorted cells were counted (Logos Biosystems, LUNA-FL), and 10000 live cells were partitioned into droplets for single-cell omics assays via Chromium Next GEM Single-Cell 5’Kit v2 (10× Genomics, 1000263). RNA-seq libraries were prepared according to the manufacturer's protocols. All libraries were quantified via the Qubit dsDNA HS Assay Kit (Invitrogen, Q32851), quality-checked for fragment sizes via High Sensitivity D5000 ScreenTapes (Agilent, 5067–5592), pooled, and sequenced (Illumina, NovaSeq 6000). The raw scRNAseq data were preprocessed using the Cell Ranger software (version 7.1.0; 10× Genomics). Feature-barcode matrices were obtained after aligning reads to a custom GRCh38 human reference genome with the CAR sequence. Sample count matrices were imported into R version 4.3.0 for further analysis in Seurat version 5.1.0^80^]. Cells with more than 15% mtRNA, fewer than 500 identified features, and more than 50,000 RNA reads were filtered out. Cell cycle state was computationally identified and regressed using Seurat’s CellCycleScoring and ScaleData functions. Doublets were computationally identified and removed using the DoubletFinder algorithm.^81^ Filtered matrices were normalized and centered, and principal component analysis was applied. The harmony algorithm^82^ was used to remove donor- and treatment-based effects in dimensional reduction. Uniform manifold approximation and projection (UMAP) dimensional reduction was applied to the first 20 principal components. Graph-based clustering was performed on the reduced data to generate distinct Seurat objects for CD4^+^ and CD8^+^ CART cells. Differential expression analysis was performed using the Seurat FindMarkers function with the Wilcoxon test and Bonferroni *P* value correction. Pseudobulk GSEA was performed by first applying Seurat’s FetchData function to procure count matrices, ranking genes according to the Wald statistic in DESeq2, and then running the cluterProfiler implementation of GSEA against the MSigDB’s HALLMARK database.

### Bulk RNAseq

Single-cell suspensions of vehicle or venetoclax-treated CART of three healthy donors were prepared and stained with Zombie NIR Dye, CD4, CD8, CAR^+^ GFP to sort CD4^+^ CAR ^+^, CD8^+^ CAR ^+^ live T cells. RNA was extracted from ~ 0.8 × 10^6^cells using RNeasy Micro Kit (Qiagen). RNA was quantified via the Qubit RNA HS Assay Kit (Invitrogen, Q32852), and quality-assessed for integrity via High Sensitivity RNA ScreenTape (Agilent, 5067–5579). 500 ng high-quality RNA with a RIN score > 7 of each sample was used for library preparation using NEBNext Ultra II Directional RNA Library Prep Kit (New England Biolabs, E7760S). Libraries were prepared according to the manufacturer's protocols. All libraries were quantified via the Qubit dsDNA HS Assay Kit (Invitrogen, Q32851), quality-checked for fragment sizes via High Sensitivity D5000 ScreenTapes (Agilent, 5067–5592), pooled, and sequenced (Illumina, NovaSeq X). RNA-sequencing reads were processed with the nf-core/rnaseq pipeline,^83^ with default settings on the University of Chicago CRI’s Randi HPC. All analysis was performed in R version 4.3.0. Gene counts were imported to the R/Bioconductor package DESeq2^84^ for differential expression analysis. Differential expression analysis was performed using DESeq2, and differentially expressed genes were identified by Benjamini–Hochberg adjusted *P* values less than or equal to 0.05 and log2FoldChange greater than 0.58 (corresponding to a 1.5-fold change in expression). Functional enrichment analysis was performed using R/Bioconductor package clusterProfiler^85,86^ with functions for GSEA (fGSEA implementation)^87,88^ and overrepresentation analysis (enricher) in reference to MSigDB’s HALLMARK database^89^ and the Kyoto Encyclopedia of Genes and Genomes (KEGG).^90^ Heatmap visualizations of *Z*-score-scaled expression are based on TPM normalization of counts.

### Statistical analysis

Data analysis was performed using GraphPad Prism V.10. All in vitro data presented are representative of at least two independent experiments except for scRNAseq (performed once with three biological replicates). Each human CART data set shows at least 2–3 different donors. All comparisons between two groups were performed using a Student’s *t*-test. Comparisons among multiple groups were evaluated with one-way ANOVA followed by Tukey’s multiple testing correction. All results are represented as mean ± SD unless otherwise noted. Survival data were analyzed using the log-rank (Mantel–Cox) test. Data analysis was performed using GraphPad Prism v10. *P* values > 0.05 were considered statistically significant.

## Supplementary information

Supplementary Material

## Data Availability

Raw scRNAseq data are provided on the Gene-Expression Omnibus (GEO) using the accession numbers GSE288296 and GSE288434. Other data generated from this study are available from the corresponding author upon reasonable request. Patient-related data not included in the paper were generated as part of clinical trials and may be subject to patient confidentiality.
