# Supplementary material for: Therapeutic targeting of BCL-2 during CART cell production augments potency through non-apoptotic adaptive changes
Source: Signal Transduct Target Ther. 2026 Apr 27;11:155. doi: 10.1038/s41392-026-02655-y (PMC13111737; doi:10.1038/s41392-026-02655-y)
Supplement: Supplementary file 1 — Supplementary Material [file 41392_2026_2655_MOESM1_ESM.docx]

Supplementary Materials for

Therapeutic Targeting of BCL-2 During CART Cell Production Augments Potency Through Non-Apoptotic Adaptive Changes

Nada S. Aboelella^1*^, Ryan Park ^1^, Erting Tang ^2^, Nicholas Asby^2^, Joshua D. Ho^1^, Tony Pan ^2^, Sidney Wang^3^, Lishi Xie^3^, Justin P. Kline^3^, Peter A. Riedell^3^, Jun Huang ^2^, James L. LaBelle^1^ ^*^

Correspondence to: [naboelella@bsd.uchicago.edu](mailto:naboelella@bsd.uchicago.edu), [jlabelle@bsd.uchicago.edu](mailto:jlabelle@bsd.uchicago.edu)

**This PDF file includes:**

Figures S1 to S10

Uncropped Western blot Figures S11 to S13

Tables S1 to S2

Figure S1

**Supplementary Fig. S1: Murine T cells treated with venetoclax *in vivo* or *in vitro* show enhanced CART cell cytotoxicity. a** Schema for *in vivo* treatment with venetoclax, CD45.1 mice were either treated with vehicle or venetoclax 50mg/kg five times/week for three weeks. Splenic T cells were isolated and activated overnight with CD3/CD28 stimulation. Cells were then transduced with murine CD19CAR constructs and treated *ex vivo* with vehicle or venetoclax (800nM). **b, c** Venetoclax treatment, either *in vivo*, *ex vivo,* or both resulted in enhanced antitumor efficacy of mCD19CARTs against CD19^+^ murine lymphoma (A20). *Ex vivo* venetoclax-treated mCD19CART showed no differences in (**d**) CD4:CD8 ratio (**e**) or T cell memory phenotype. T_N_: naïve (CD62L^+^CD44^-^), T_CM_: central memory (CD62L^+^CD44^+^), T_EM_: effector memory (CD44^+^CD62L^-^). Data are from three biological replicates (n=3) and shown as mean ± SD. One-way ANOVA followed by Tukey’s multiple comparison tests was used for (**c**), unpaired Student’s t-test for (**d**) and two-way ANOVA followed by SIDAK's multiple comparison tests for (**e**). *P < 0.05, **P<0.01, ***p<0.001, ns: non-significant. Image (**a**) created in BioRender.

Figure S2

**Supplementary Fig. S2: Venetoclax-treated CART killing is antigen specific and is not dependent on the CAR costimulatory component or cytokines used during manufacturing. a** CD19 expression of OCI-Ly8.CD19KO (gray) compared to parental OCI-Ly8 (red). **b** Vehicle-treated and venetoclax-CART show negligible (non-specific) killing of OCI-Ly8 indicating a dependence on CD19 for specificity. **c** Venetoclax augments the antitumor efficacy of both CD28 and 4-1BB containing CART cells *in vitro.* **d** Venetoclax-mediated CART enhancement is cytokine independent. CART manufactured using IL-2 or IL-7/15 showed no difference in the ability or venetoclax to enhance their killing capacity *in vitro*. One-way ANOVA with post-hoc Tukey’s tests was performed for (**b-d**). *P < 0.05, *** P < 0.001, and ****p<0.0001, ns: non-significant.

**Figure S3**

**Supplementary Fig. S3: Venetoclax-treated** **CART have greater effector cytokines, utilize TRAIL to kill targets, and have greater persistent antitumor capacity compared to vehicle-treated CART. a** Venetoclax enhances the effector function of CART through dose-dependent increases in effector cytokines and granzyme B following tumor recognition. **b** Venetoclax - induced TRAIL expression is partially responsible for the antitumor activity of venetoclax-treated CART as measured via TRAIL neutralization. **c** Schema for serial killing assay. CART were cultured with luciferase expressing OCI-Ly8 tumors in E:T ratio of 1:1 and tumor signal was monitored by live cell imagining. T cells were harvested and counted when no tumor signal was detected, then re-plated with fresh OCI-Ly8 tumor cells at an E:T ratio of 1:1. **d** Venetoclax maintains greater CART activity against tumor after three rounds of tumor challenge compared to vehicle-treated CART while having the same level of proliferation. Image (**c**) created using SMART - Servier Medical ART.

**Figure S4**

**Supplementary Fig. S4: Venetoclax** **induces transcriptional alterations in the CART product. a** Multidimensional principal component analysis (PCA) of gene expression from vehicle or venetoclax-treated CD4^+^ and CD8^+^ CART cells. **b** Transcriptional profiles of CD4^+^ and CD8^+^ CART cells by HALLMARK analysis of significantly upregulated (red) and downregulated (blue) pathways. **c** Gene set enrichment of RNA sequencing data from CD4^+^ and CD8^+^ CART cells using the Molecular Signatures Database (MSigDB). **d** Venetoclax induces significant transcriptional changes in BCL-2 family genes in CD4^+^ and CD8^+^ CART. Gene expression of venetoclax-treated CART is normalized to vehicle-treated CART.

**Figure S5**


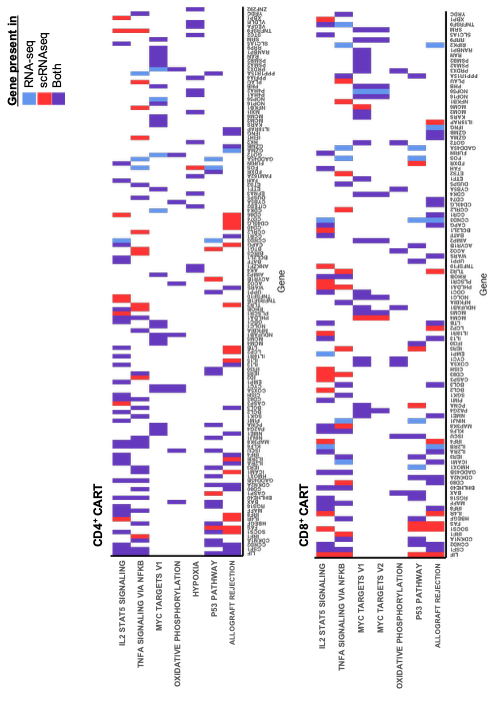


**Supplementary Fig. S5: Shared gene set enrichment analyses from bulk and single-cell RNAseq of venetoclax-treated CART.** Data represent those from vehicle or venetoclax-treated CART prepared from six healthy donors (bulk RNAseq n=3 (light blue) and scRNAseq n=3 (red)) and pooled from two separate experiments. The most frequent leading-edge genes across common significantly enriched pathways from bulk RNAseq and scRNAseq (purple) GSEA against HALLMARK pathways are shown.

Figure S6

**Supplementary Fig. S6: STAT5 pathway activation, and not ROS generation, is responsible for the increased killing efficacy of venetoclax-treated CART. a** Therapeutic inhibition of STAT5 with AC-4-130 reverses the increased killing effect following venetoclax treatment as measured using *in vitro* tumor killing assays against OCI-Ly8 DLBCL cells. Effector to tumor (E:T) ratio of 1:5 and incubation for 48 hours prior to imaging. **b** Westen blot of total and phosphorylated forms of STAT5 and AKT in addition to BCL-2, BCL-X_L_, and MCL-1 in vehicle vs venetoclax-treated CART at baseline or in experiments where inhibitors of STAT5 (STAT5i) or AKT (AKTi) were used. **c** Incubation of venetoclax-treated CART with L-N-Acetyl Cysteine (LNAC) has no effect on their tumor killing capacity. **d** STAT5 inhibition (STAT5i) significantly decreases basal respiration, maximal respiration, space respiratory capacity (SRC), glycolysis, glycolytic capacity and reserve in venetoclax-treated CART while AKT inhibition (AKTi) equally decreases all parameters in both vehicle- and venetoclax treated CART. Seahorse data represent analysis of n = 8 technical replicates. One-way ANOVA followed by Tukey’s multiple comparison tests was used for (**d**).

**Figure S7**

**Supplementary Fig. S7: Venetoclax affects the expression of key metabolic genes in venetoclax-treated CART.** Treatment-wise expression distributions of selected metabolic genes in whole (CD4^+^/CD8^+^) CART products measured using scRNAseq. Expression level reflects log-normalized counts. **a** Downregulated genes involved in glycolysis and (**b**) upregulated genes associated with fatty acid metabolism in venetoclax-treated CART compared to vehicle-treated CART.

**
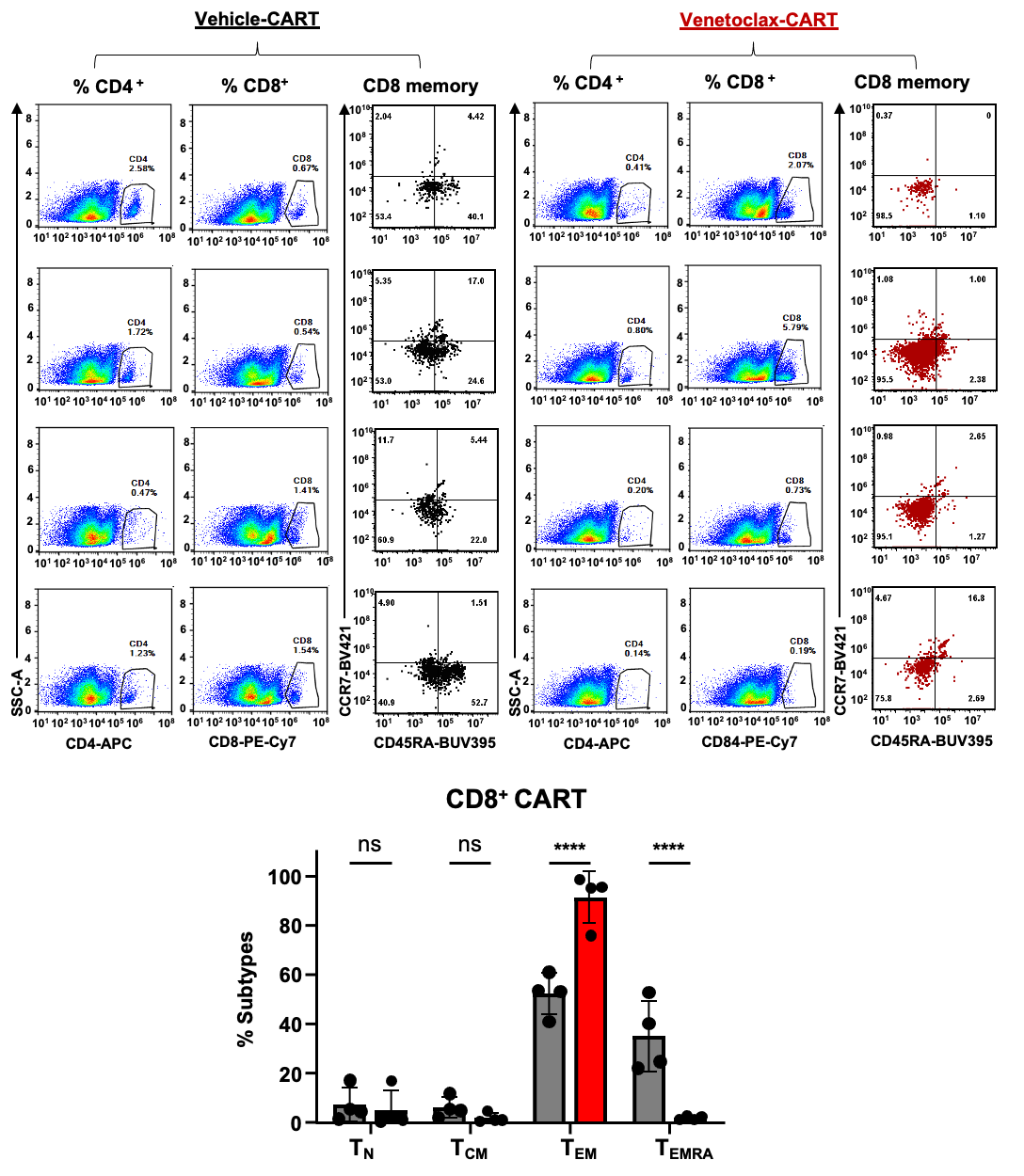
Figure S8**

**Supplementary Fig. S8: Venetoclax enhances the *in vivo* accumulation of effector CD8^+^ CART.** Following the experiment procedure in Fig. 6A, mice with NALM6 tumors treated with vehicle or venetoclax-treated CART were sacrificed on day 24 after CART infusion. Spleen analysis indicated that mice treated with venetoclax-treated CART significantly maintain CART at an effector memory phenotype compared to terminally differentiated phenotype of vehicle-treated CART.

**Figure S9**

**Supplementary Fig. S9: Venetoclax enhances the *in vitro* potency of CART prepared from patient samples regardless of their starting T cell composition, memory phenotype, or exhaustion profiles.** Patient-derived PBMCs (PT-35 and PT-51) collected at the time of commercial CART cell apheresis were used to prepare vehicle or venetoclax-treated CART. PBMCs from healthy volunteers were used as comparators. Starting material was evaluated for (**a**) CD4:CD8 ratio, (**b**) CD4^+^ FOXP3^+^ Treg content, (**c**) CD4^+^ and CD8^+^ T cell subpopulation content as determined by CCR7 and CD45RA, (**d**) T cells memory status based on the surface expression of CD62L and CD127, and (**e**) protein expression levels of BCL-2, BCL-X_L_, and MCL-1. **f** Venetoclax treatment had no effect on CAR expression compared to vehicle treatment in individual CART products. **g** Venetoclax-treated CART prepared from patient samples (PT-35 or PT-51) resulted in increased *in vitro* killing of OCI-Ly8 tumor cells after 48 hours of co-culture at an E:T ratio of 1:5 that was comparable to CART prepared from a healthy volunteer. Quantified results in adjacent bar graphs show that venetoclax dose-dependently enhances the anti-tumor efficacy of CART. One-way ANOVA with post-hoc Tukey’s tests was performed for (**g**). *P < 0.05, *** P < 0.001, and ****p<0.0001, ns: non-significant.


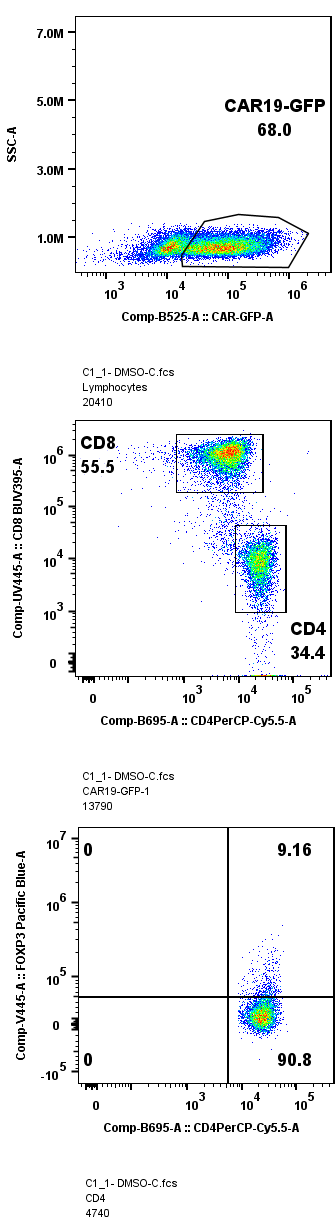

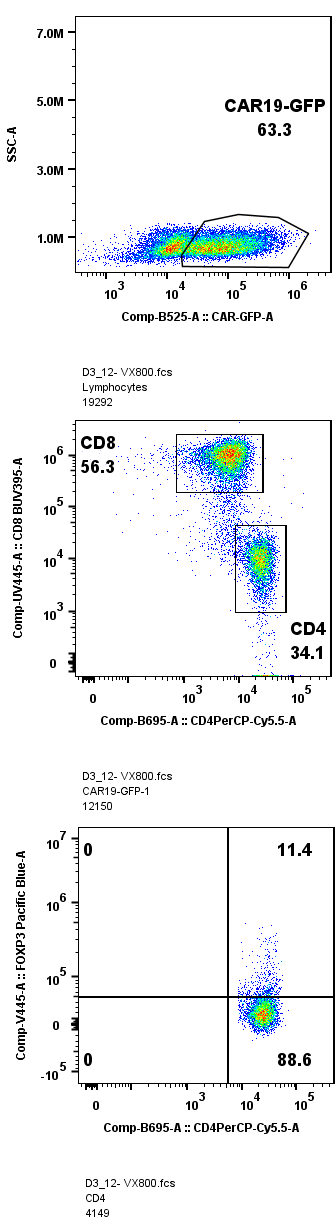

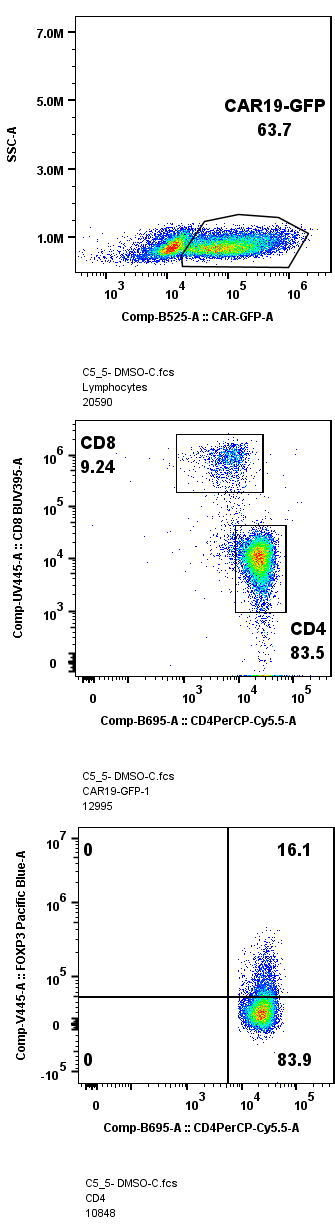

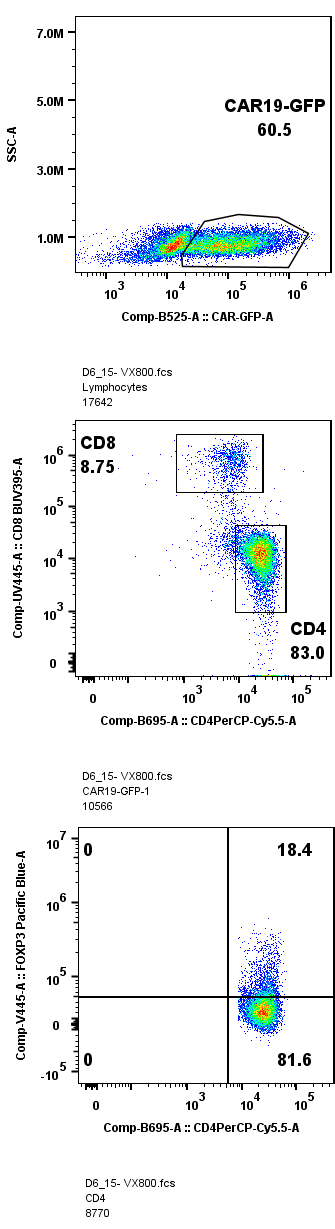

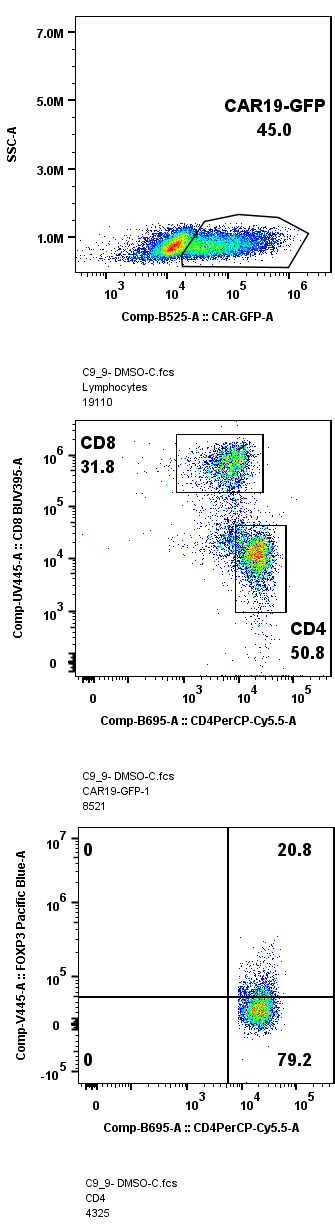

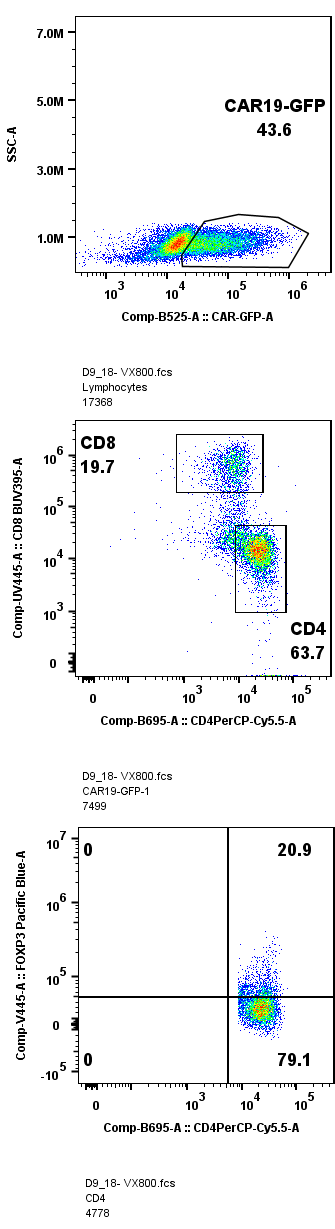

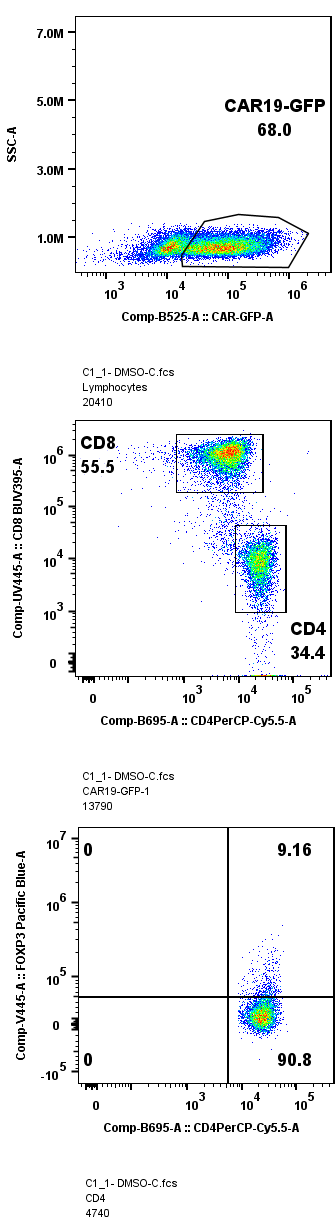

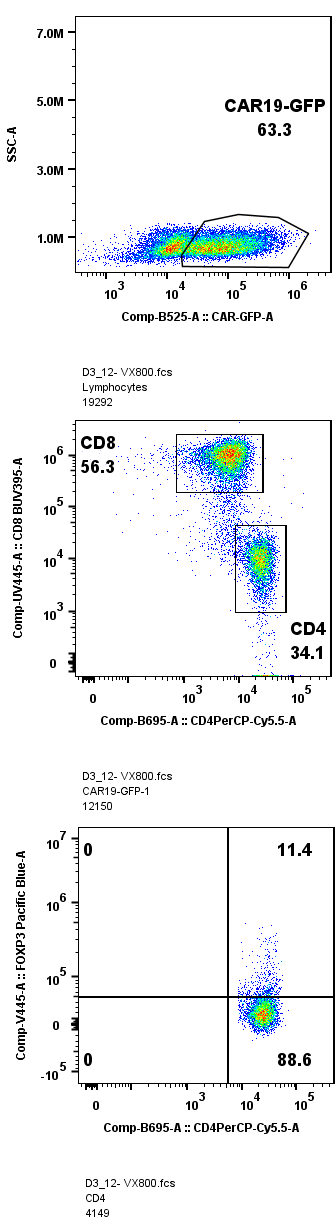

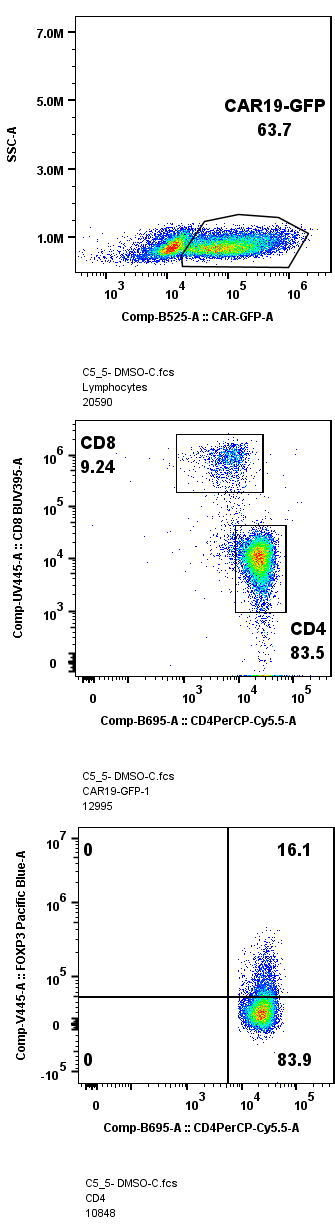

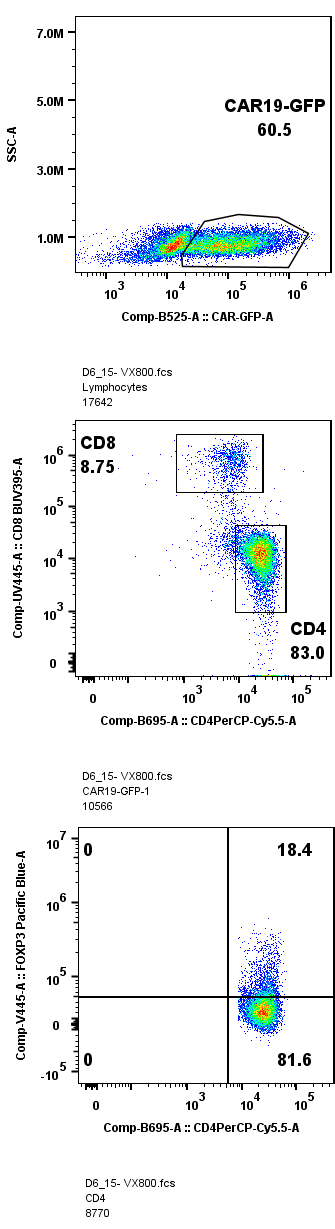

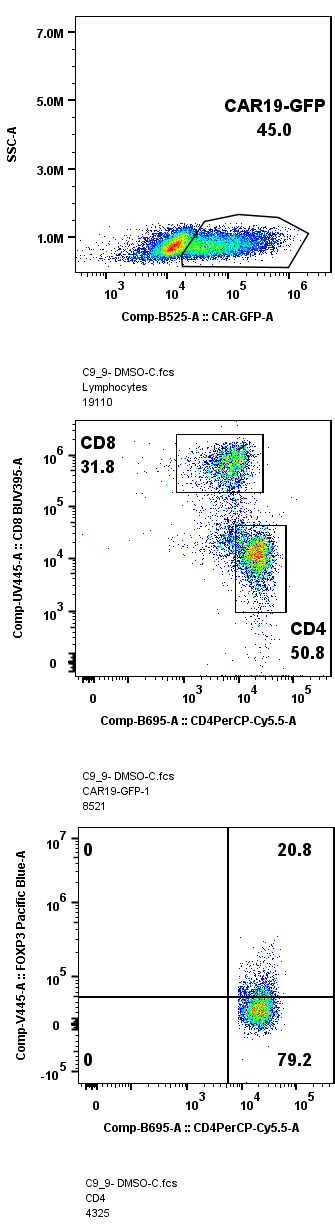

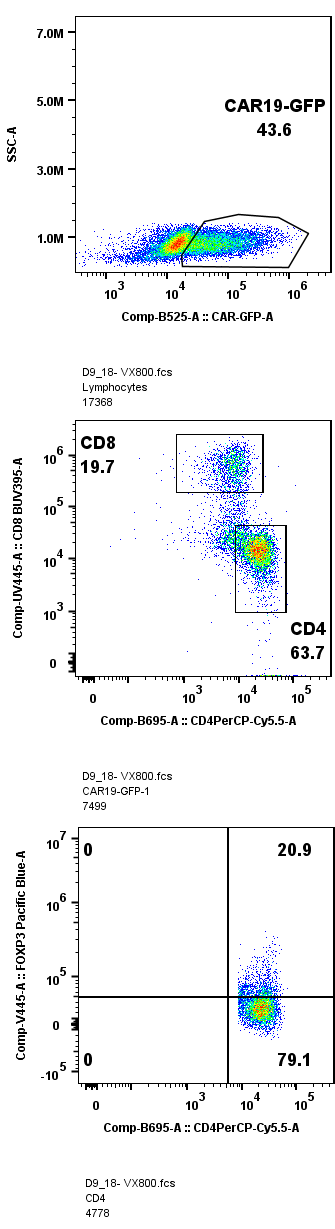

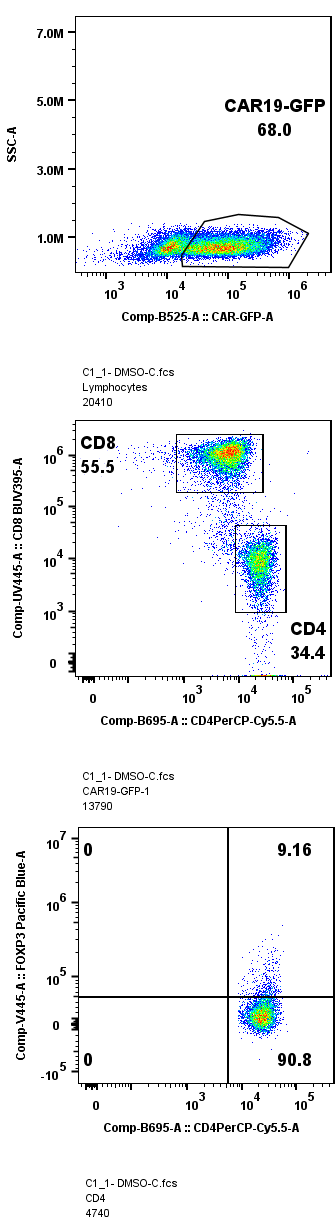

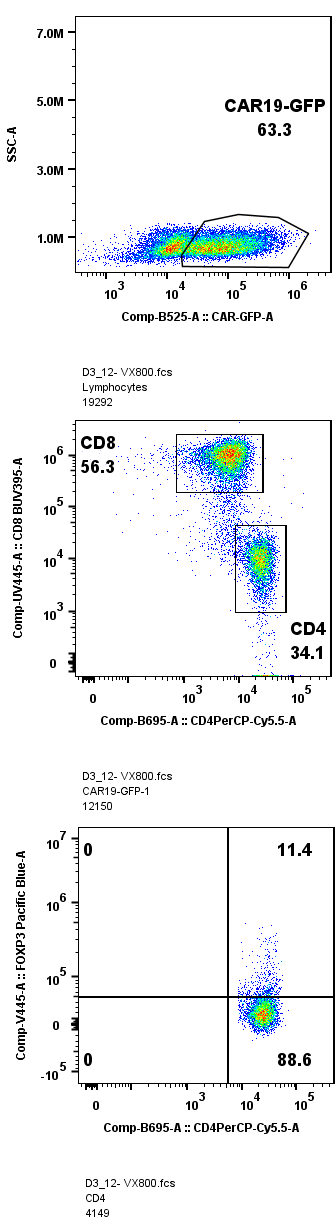

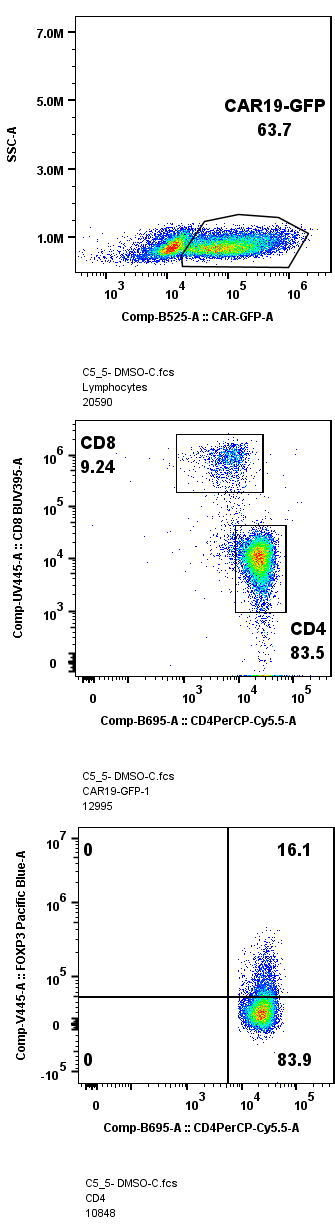

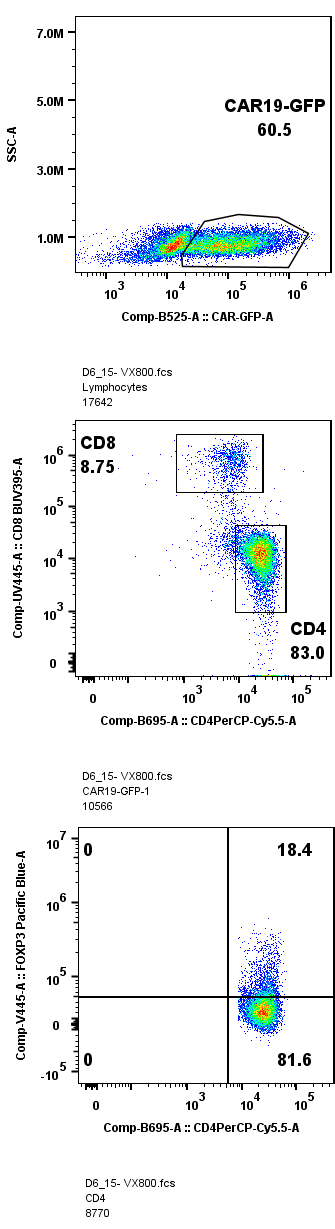

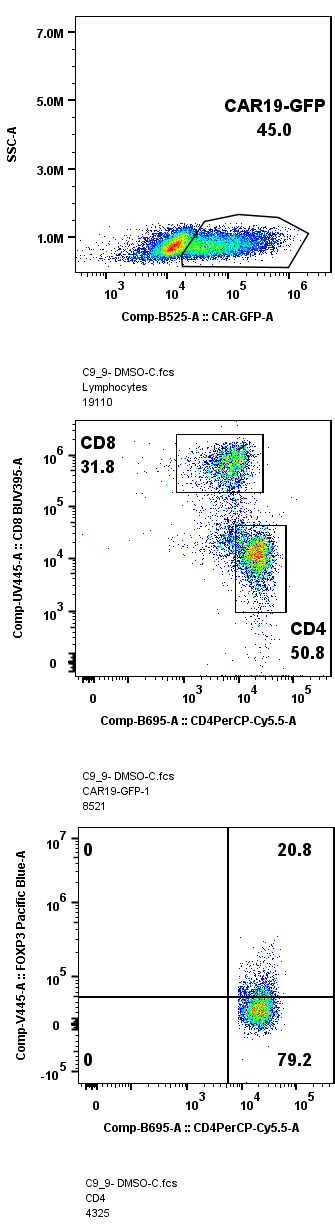

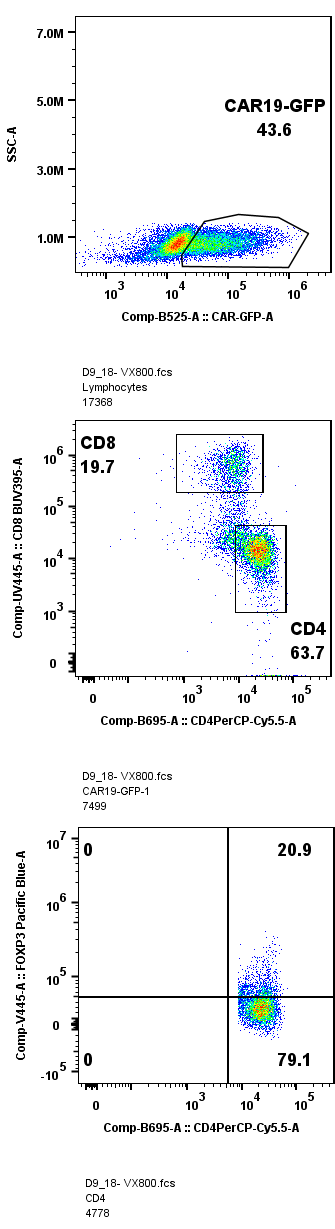
Figure S10

**Healthy**

**PT-126**

**PT-28**

**Vehicle**

**Venetoclax**

**Vehicle**

**Venetoclax**

**Vehicle**

**Venetoclax**

**b**

**c**

**FOXP3- Pacific Blue**

**CD4-PerCP-Cy5.5**

**10^3^**

**10^4^**

**10^5^**

**0**

**-10^5^**

**10^5^**

**10^6^**

**10^7^**

**0**

**10^3^**

**10^4^**

**10^5^**

**0**

**10^3^**

**10^4^**

**10^5^**

**0**

**10^3^**

**10^4^**

**10^5^**

**0**

**10^3^**

**10^4^**

**10^5^**

**0**

**10^3^**

**10^4^**

**10^5^**

**0**

**CD8-BUV395**

**10^3^**

**10^4^**

**10^5^**

**0**

**10^3^**

**10^5^**

**10^6^**

**10^4^**

**0**

**CD4-PerCP-Cy5.5**

**10^3^**

**10^4^**

**10^5^**

**0**

**10^3^**

**10^4^**

**10^5^**

**0**

**10^3^**

**10^4^**

**10^5^**

**0**

**10^3^**

**10^4^**

**10^5^**

**0**

**10^3^**

**10^4^**

**10^5^**

**0**

**10^3^**

**10^4^**

**10^5^**

**10^6^**

**1**

**3**

**5**

**7**

**CD19CAR-GFP**

**SSC-A**

**a**

**Supplementary Fig. S10: Venetoclax has no effect on CAR expression in CD4^+^ or CD8^+^ CART cell compartments within patient sample-derived products. A** CAR transduction of vehicle- and venetoclax-treated T cells from the healthy volunteer and two patients (PT-28 and PT-126) used for the studies shown in Figure 7. **b** CART prepared from the healthy donor have a higher percentage of CD8^+^ CART compared to patient-derived CART. **c** CD4^+^ FOXP3^+^ Treg CART content is lower in healthy volunteer-derived CART prepared compared to patient-derived CART.

Figure S11


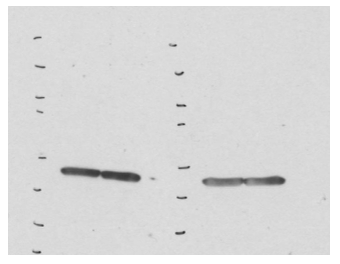

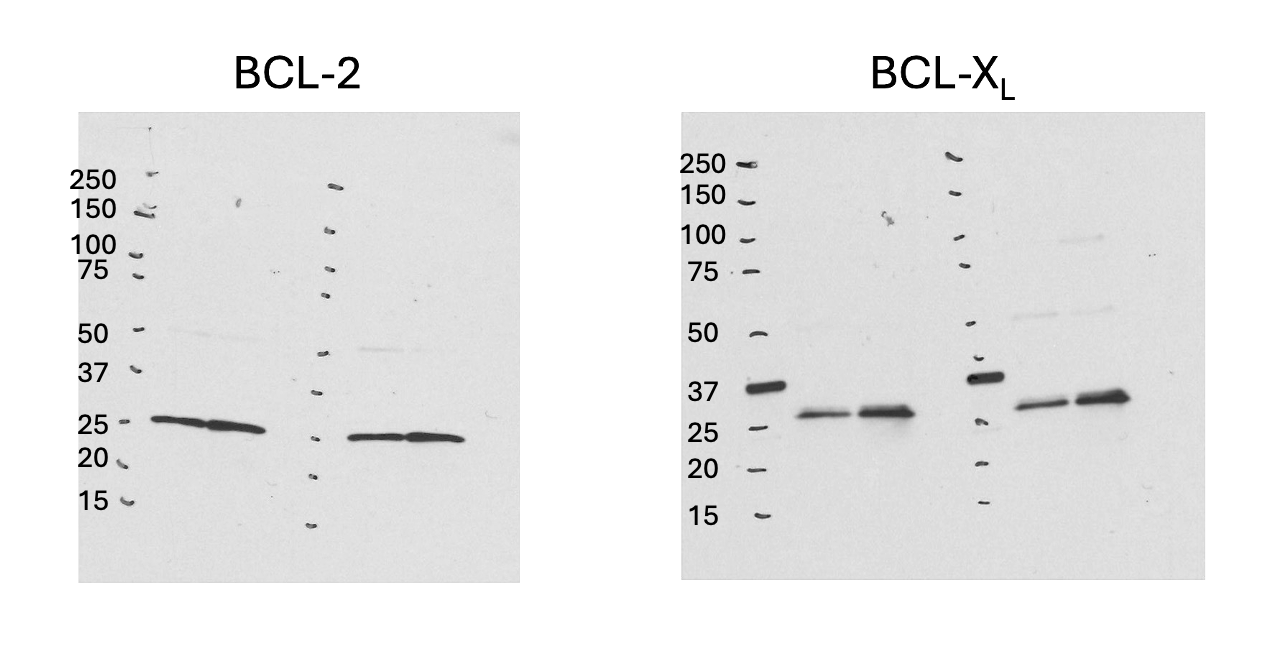

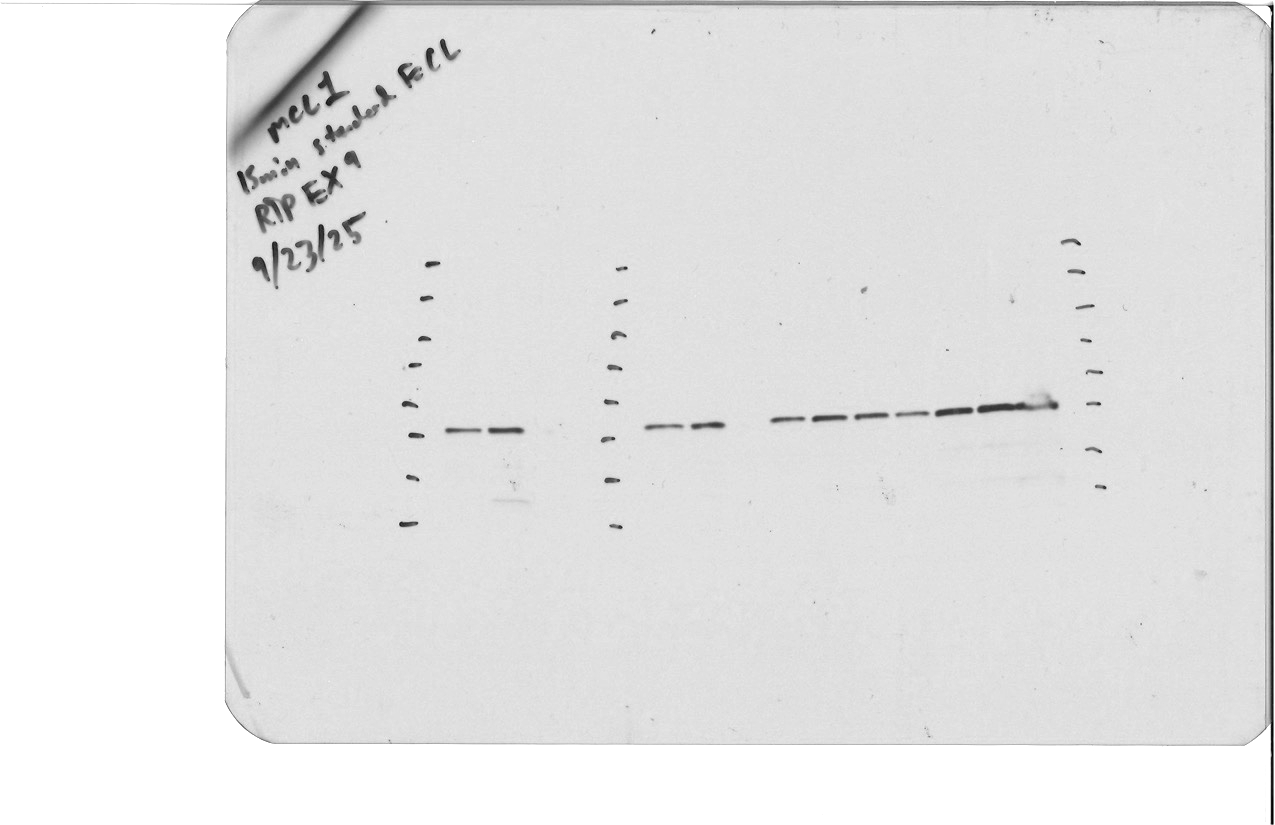

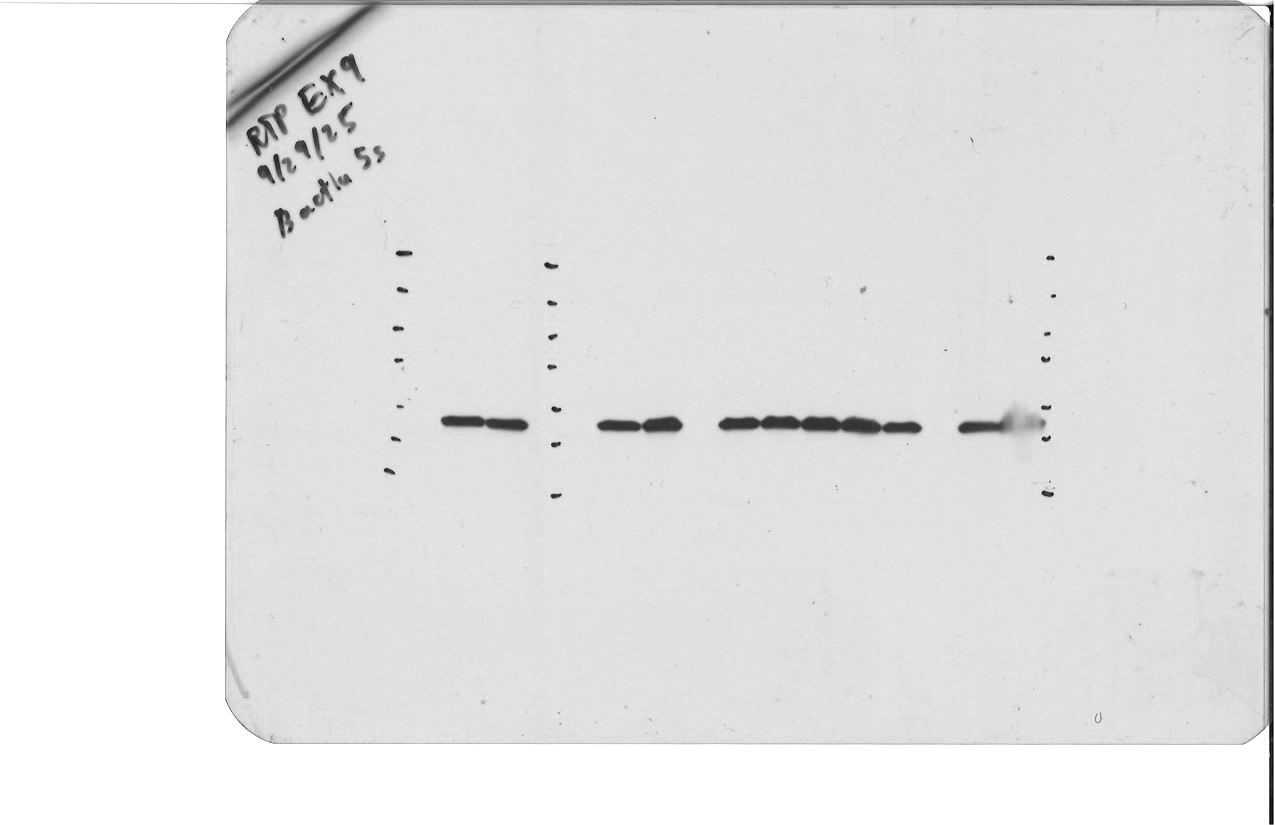

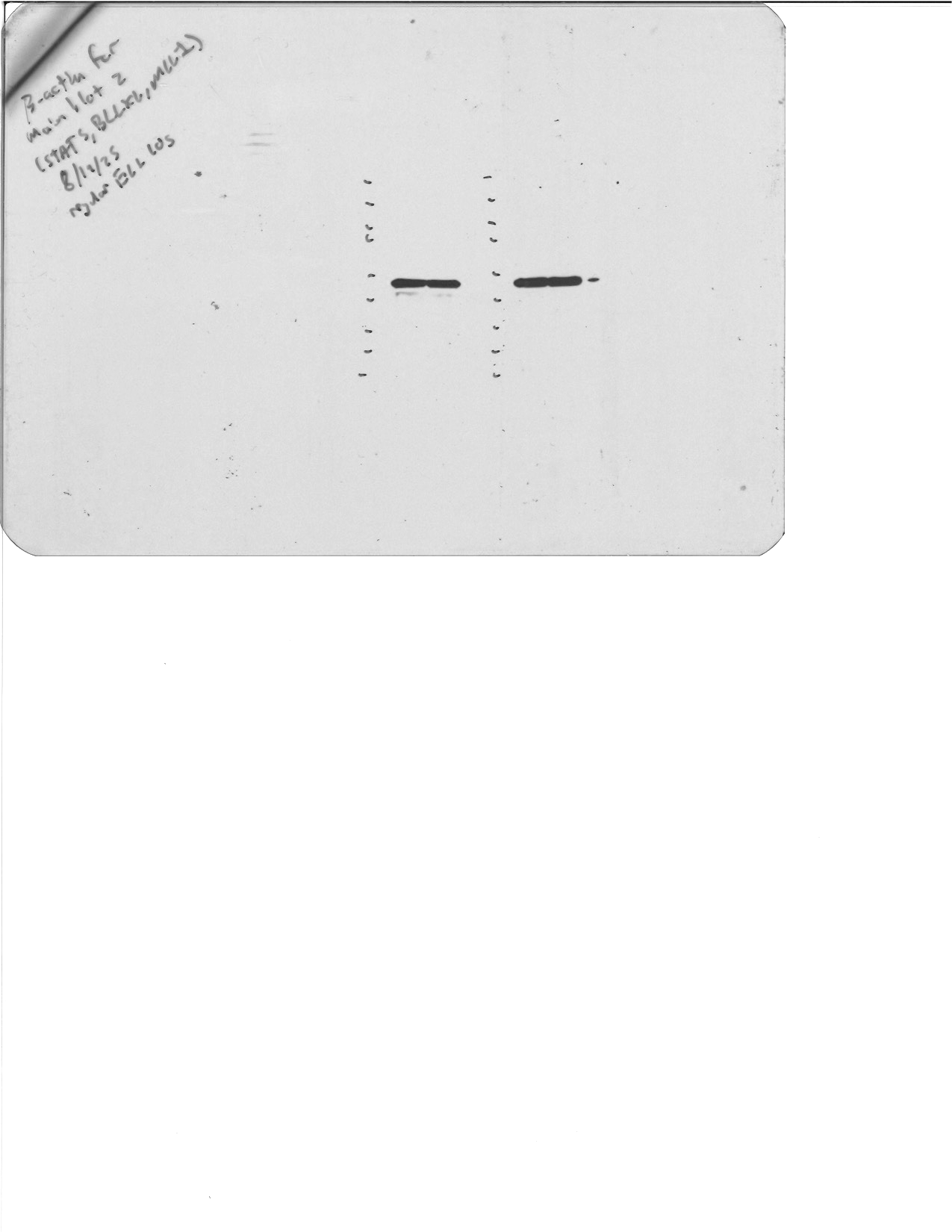


250

150

100

75

50

37

25

20

MCL-1

BCL-2

BCL-X_L_

250

150

100

75

50

37

25

250

150

100

75

50

37

25

250

150

100

75

50

37

25

Beta-actin controls

**Uncropped Western blots Figure 1b**

Figure S12


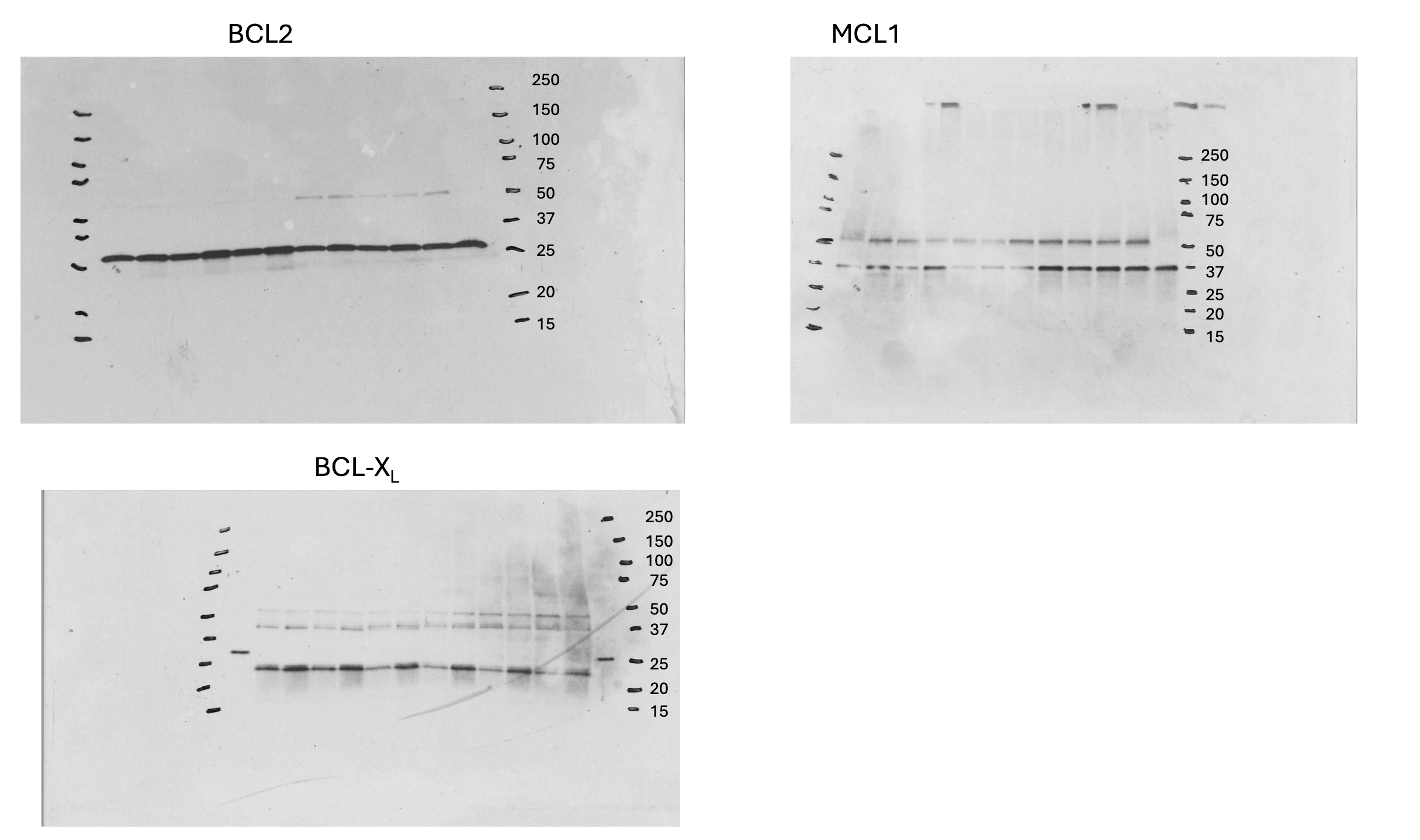

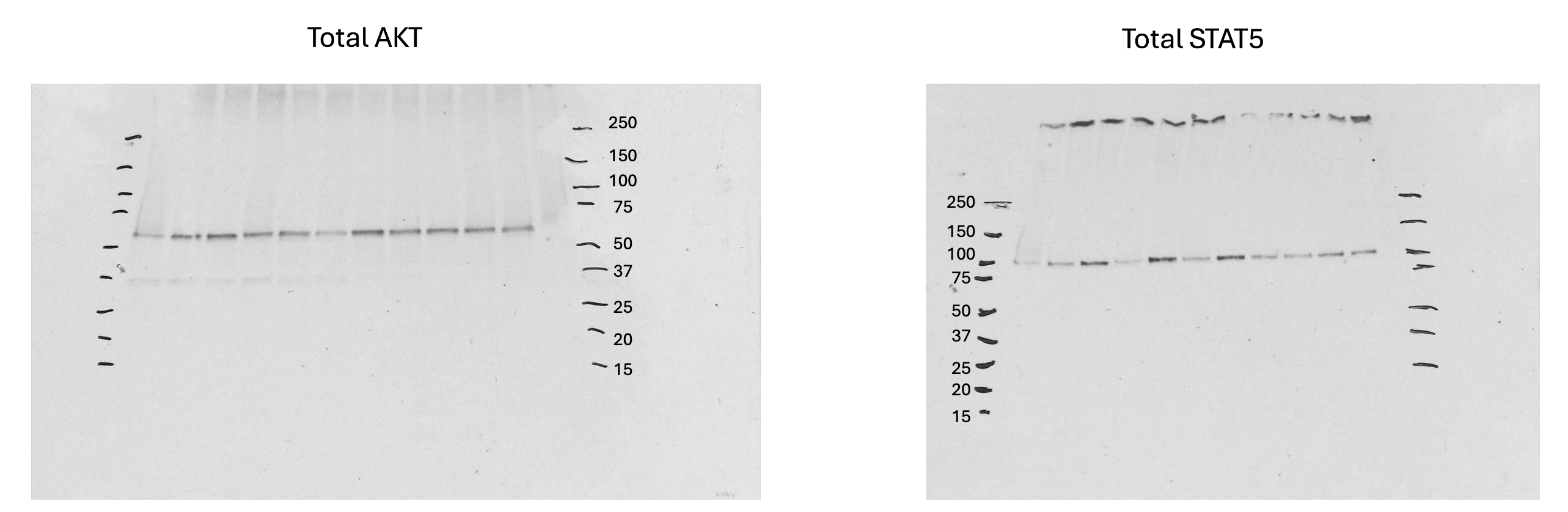

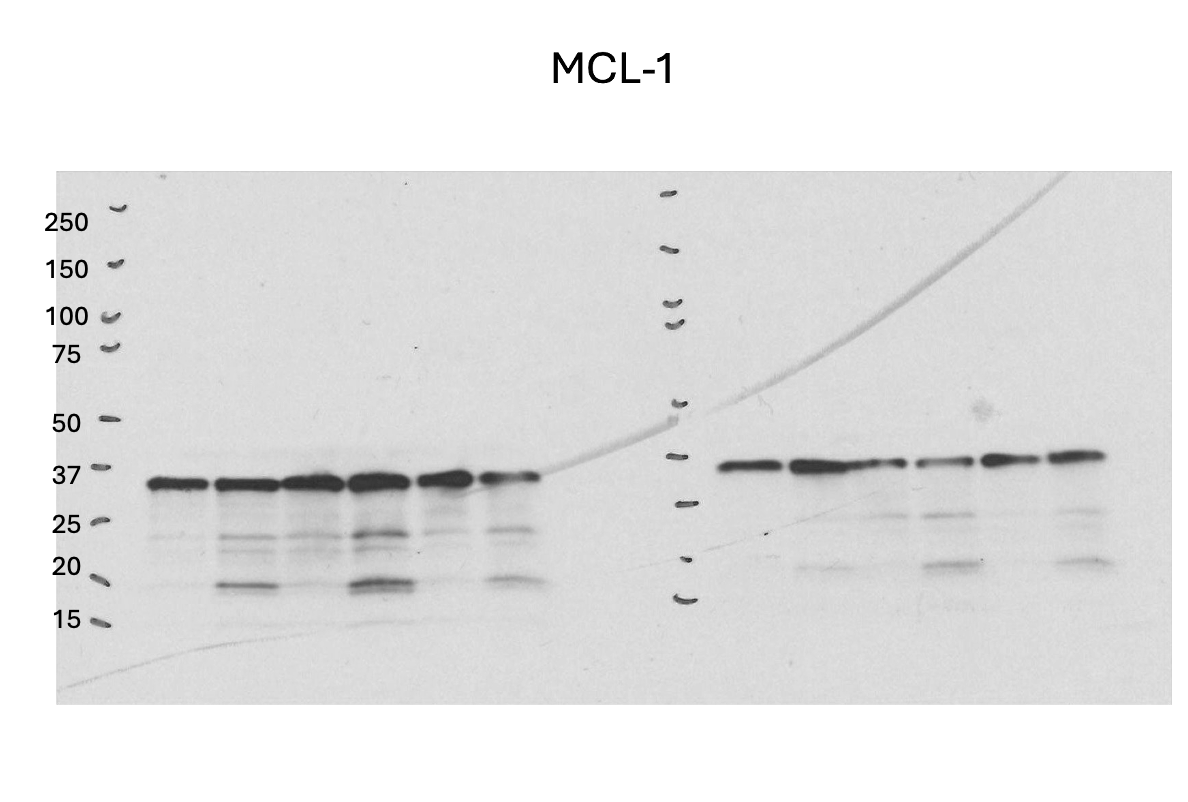

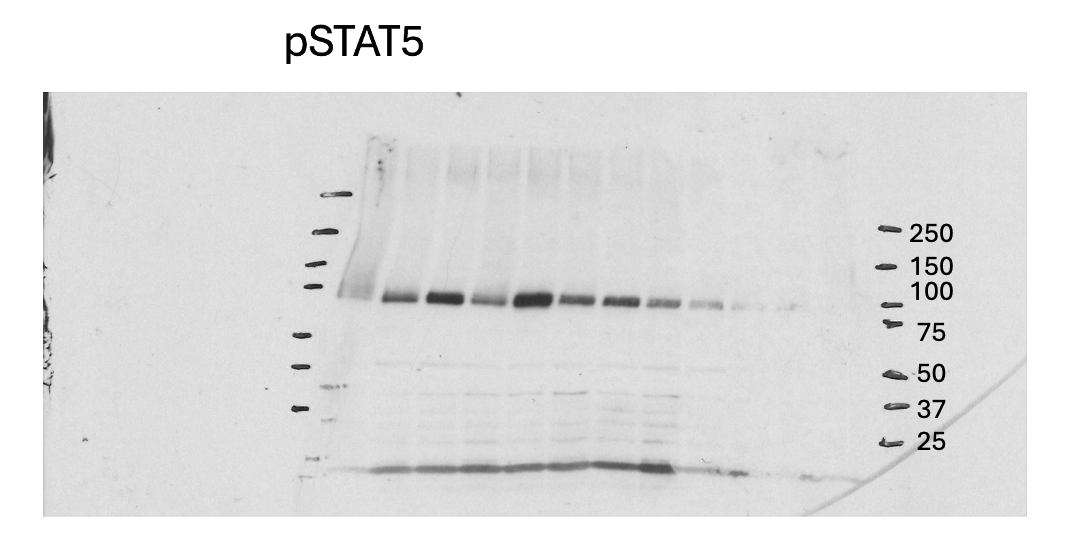

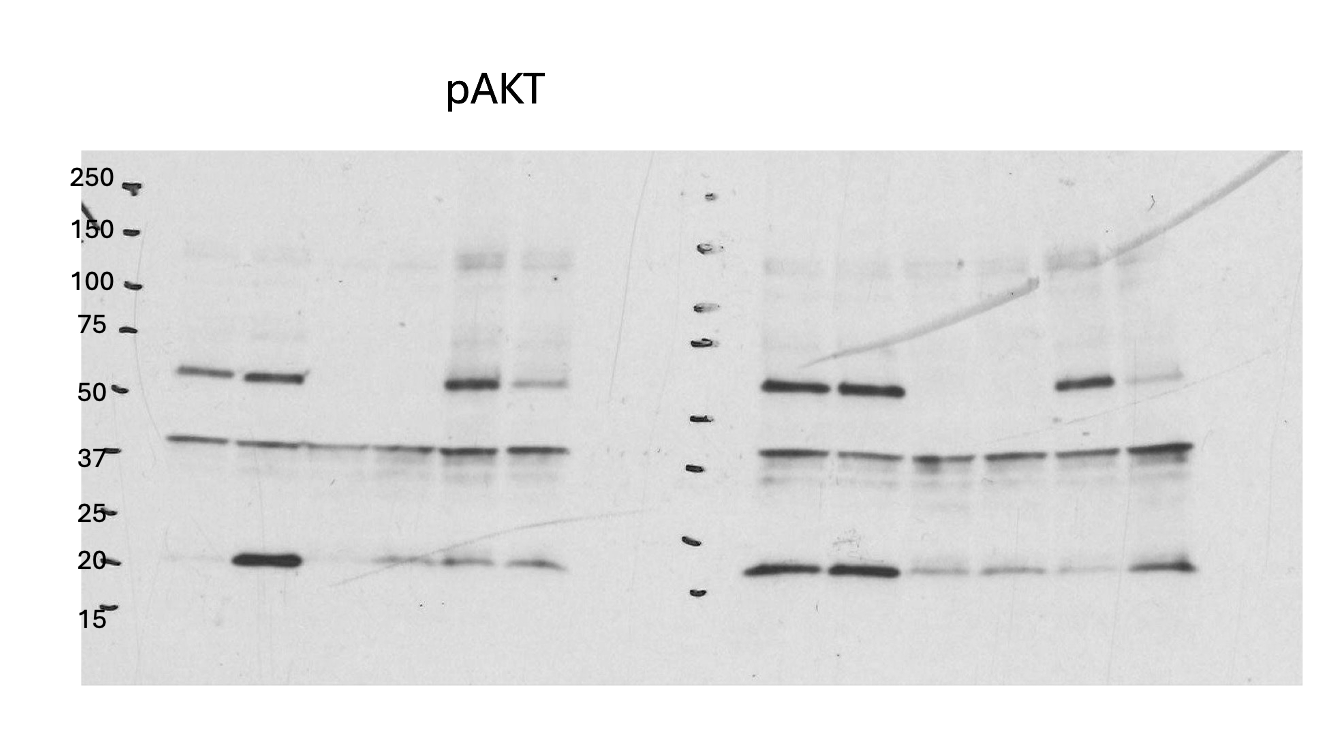


**Uncropped Western blots Supplementary Fig. S6**

Figure S13


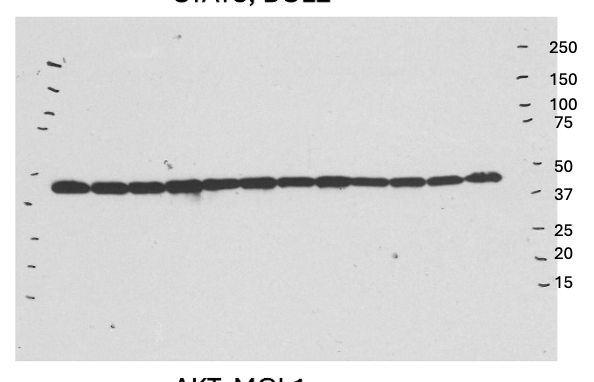

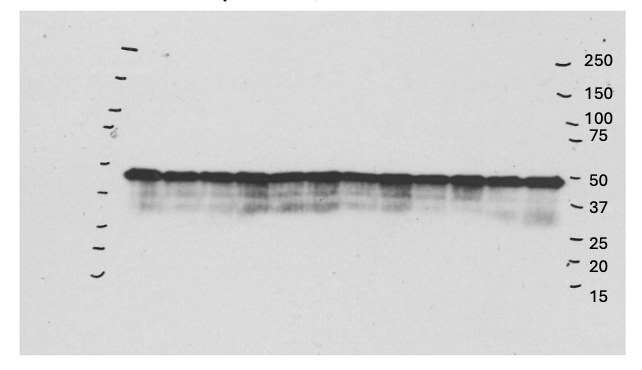

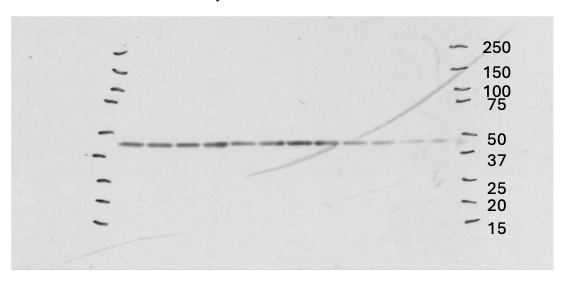

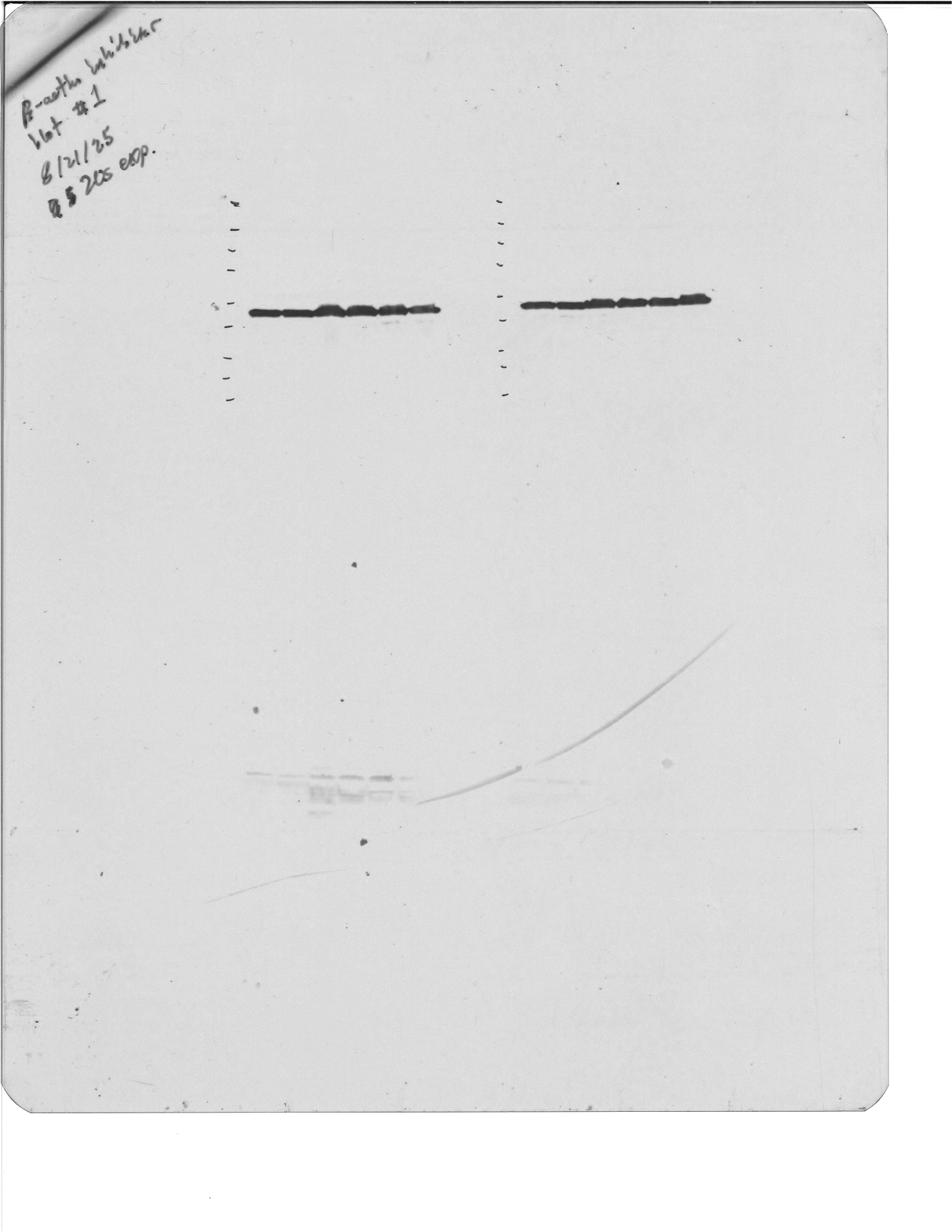


Beta-actin control for BCL2, STAT5

Beta-actin control for BCL-XL, pSTAT5

Beta-actin control for AKT

Beta-actin control for MCL-1, pAKT

250

150

100

75

50

37

25

20

15

**Uncropped Western blots Supplementary Fig. S6 (Continued)**

Table S1

| **Patient ID** | **Disease type** | **Total number of lines of prior therapy** | **Age at leukapheresis** | **Sex** |
| --- | --- | --- | --- | --- |
| **PT-28** | HGBL | 3 | 74 | Male |
| **PT-35** | HGBL | 2 | 77 | Male |
| **PT-51** | DLBCL | 2 | 70 | Male |
| **PT-126** | HGBL | 3 | 81 | Male |

**Supplementary Table S1: Patient samples used to prepare CART.**

Table S2

**Supplementary Table S2: Antibodies used for flow cytometric studies.**
